# Epithelial cell adhesion molecule (EpCAM) regulates HGFR signaling to promote colon cancer progression and metastasis

**DOI:** 10.1186/s12967-023-04390-2

**Published:** 2023-08-05

**Authors:** Chi-Chiu Lee, Chia-Jui Yu, Sushree Shankar Panda, Kai-Chi Chen, Kang-Hao Liang, Wan-Chen Huang, Yu-Shiuan Wang, Pei-Chin Ho, Han-Chung Wu

**Affiliations:** 1 Institute of Cellular and Organismic Biology, Academia Sinica, 128 Academia Road, Section 2, Nankang, Taipei, 11529 Taiwan; 2 Biomedical Translation Research Center (BioTReC), Academia Sinica, Taipei, 11529 Taiwan

**Keywords:** EpCAM, EpEX, HGFR, Cancer progression, Epithelial-to-mesenchymal transition, Invasion, Colorectal cancer

## Abstract

**Background:**

Epithelial cell adhesion molecule (EpCAM) is known to highly expression and promotes cancer progression in many cancer types, including colorectal cancer. While metastasis is one of the main causes of cancer treatment failure, the involvement of EpCAM signaling in metastatic processes is unclear. We propose the potential crosstalk of EpCAM signaling with the HGFR signaling in order to govern metastatic activity in colorectal cancer.

**Methods:**

Immunoprecipitation (IP), enzyme-linked immunosorbent assay (ELISA), and fluorescence resonance energy transfer (FRET) was conducted to explore the extracellular domain of EpCAM (EpEX) and HGFR interaction. Western blotting was taken to determine the expression of proteins in colorectal cancer (CRC) cell lines. The functions of EpEX in CRC were investigated by proliferation, migration, and invasion analysis. The combined therapy was validated via a tail vein injection method for the metastasis and orthotopic colon cancer models.

**Results:**

This study demonstrates that the EpEX binds to HGFR and induces downstream signaling in colon cancer cells. Moreover, EpEX and HGF cooperatively mediate HGFR signaling. Furthermore, EpEX enhances the epithelial-to-mesenchymal transition and metastatic potential of colon cancer cells by activating ERK and FAK-AKT signaling pathways, and it further stabilizes active β-catenin and Snail proteins by decreasing GSK3β activity. Finally, we show that the combined treatment of an anti-EpCAM neutralizing antibody (EpAb2-6) and an HGFR inhibitor (crizotinib) significantly inhibits tumor progression and prolongs survival in metastatic and orthotopic animal models of colon cancer.

**Conclusion:**

Our findings illuminate the molecular mechanisms underlying EpCAM signaling promotion of colon cancer metastasis, further suggesting that the combination of EpAb2-6 and crizotinib may be an effective strategy for treating cancer patients with high EpCAM expression.

**Supplementary Information:**

The online version contains supplementary material available at 10.1186/s12967-023-04390-2.

## Background

EpCAM is a type I transmembrane protein with 314 amino acids and an observed molecular weight of 39–42 kDa. It contains an extracellular domain (EpEX, 265 amino acids), a single transmembrane domain, and a short intracellular domain (EpICD, 26 amino acids). The domains are cleaved and released via regulated intramembrane proteolysis (RIP) by a disintegrin and metalloprotease ADAM17 (also called tumor necrosis factor-α converting enzyme, TACE) and a multi-subunit protease complex, γ-secretase [1]. RIP can trigger EpCAM-mediated signal transduction through the shedding of EpEX by ADAM17 and EpICD by γ-secretase complex [1, 2] and play important roles in tumor initiation and progression [3–6]. Although, EpCAM is absent or weakly expressed in the vast majority of healthy epithelial squamous cells, it is strongly expressed in squamous cell carcinomas [7]. Furthermore, the expression of EpCAM in squamous carcinomas is correlated with increased cellular proliferation and decreased differentiation [8]. Our group previously developed a neutralizing antibody against EpCAM, EpAb2-6, which has strong potential for use as a colorectal carcinoma (CRC) treatment [5, 9, 10]. Despite its promise as a therapeutic target in CRC, the mechanisms through which EpCAM contributes to tumorigenesis and metastasis are still not completely known.

HGFR (hepatocyte growth factor receptor), also called c-Met, is a high affinity receptor tyrosine kinase (RTK) that is activated by hepatocyte growth factor (HGF, also known as Scatter Factor) and encoded by the *MET* gene [11]. The tyrosine kinase domain of HGFR contains two tyrosine residues at positions 1234 and 1235, and the phosphorylation of these two sites is essential for activating the HGFR receptor [12]. Many reports have demonstrated critical roles for HGFR in tumorigenesis, cell growth, survival, and metastasis [13, 14]. Hepatocyte growth factor (HGF), the ligand of HGFR, is a pleiotropic cytokine mainly produced by mesenchymal cells, including fibroblasts and macrophages. The activation of HGFR can induce through HGF binding to HGFR resulting in HGFR homodimerization, or HGFR dimerizes with different receptor pathways [15]. HGFR activation induces tumor progression via signaling cascades that mainly affect the tumor angiogenesis, growth, motility, and metastasis in many cancer cells, including colorectal cancer (CRC) [16, 17]. HGFR overexpression has been reported in CRC, which has been demonstrated to be critically attributable to CRC stemness and poor prognosis [18, 19]. Clarifying the mechanism of regulating HGFR signaling in CRC is important for finding effective therapy for CRC.

Epithelial–mesenchymal transition (EMT) is associated with tumorigenic process and metastasis [20]. Cancer cells that undergo EMT exhibit enhanced cell motility and invasion through the induction of mesenchymal properties and loss of epithelial cell adhesion. Indicators of EMT include increased expression of mesenchymal markers, such as Vimentin, Snail, and Slug, along with decreased expression of epithelial markers like E-cadherin [21]. In addition, many RTKs including HGFR signaling, promote the EMT program, thereby enhancing the invasive and metastatic potential and drug resistance of cancer cells [22].

EpEX contains two epidermal growth factor (EGF)-like domains, and it may serve as a soluble growth factor to activate EGF receptor (EGFR) in the local tumor microenvironment [5, 6, 9]. A previous report showed that activation of EGFR could trigger RIP of EpCAM to induce EMT [23]. Of note, EGFR is a RTK that is highly relevant in many types of cancer since it is overexpressed in a variety of tumors [24]. Furthermore, EGFR-dependent phosphorylation and activation of HGFR have been reported to occur upon stimulation of epidermal carcinoma cells with EGFR ligands [25]. In addition, such cross-activation of HGFR in cells with elevated EGFR signaling has also been observed in several types of tumors [26].

In this study, we show that EpEX binds to HGFR and activates its downstream signaling to promote cell proliferation, migration, and invasion. Furthermore, these effects are mediated by the regulation of Snail protein stability. We also found that our EpCAM neutralizing antibody, EpAb2-6, attenuates the phosphorylation of HGFR and inhibits cancer cell metastasis. Thus, the results of this study provide a mechanistic rationale for the simultaneous targeting of EpCAM and HGFR signaling to combat metastasis in CRC.

## Material and methods

### Chemicals and antibodies

Anti-α-tubulin and GAPDH antibodies were from Sigma-Aldrich. Antibodies against human EpCAM, total ERK and Thr202/Tyr204-phosphorylated ERK, total AKT, Ser473-phosphorylated AKT, total HGFR, Tyr1234/1235-phosphorylated HGFR, Non-phospho (Active) β-Catenin (Ser45), β-Catenin, E-cadherin, Vimentin, Snail, Slug, and Twist were from Cell Signaling Technology. LY294002 (AKT inhibitor) was also from Cell Signaling Technology. SU11274 (HGFR inhibitor), Crizotinib (HGFR inhibitor), U0126 (MEK inhibitor), PF-562271 (FAK inhibitor), TAPI-1 (ADAM17 inhibitor), DAPT (γ-secretase inhibitor), and BIO (GSK3 beta inhibitor) were obtained from Selleck Chemicals. Crizotinib (HGFR inhibitor) was obtained from Med Chem Express. Antibodies against total GSK3 beta, phosphorylated GSK3 beta (phospho S9), phosphorylated ADAM17 (phospho T735), total ADAM17, phosphorylated presenilin 2/AD5 (phospho S327), total presenilin 2/AD5, V5-tag, 6× His-tag, and c-Myc-tag, as well as the Met (pY1234/pY1235) + total Met ELISA Kit (ab126451) were obtained from Abcam. Human HGFR (c-MET) and HGF recombinant proteins were obtained from Sino Biological. The four mutant Snail constructs were obtained from Addgene.

### Cell lines and culture

The following human cell lines were used: HEK293T, colorectal cancer cell line HCT116 (ATCC: CCL-247), and HT29. The cells were cultivated in Dulbecco modified Eagle’s media (DMEM) supplemented with 10% fetal bovine serum (FBS; Gibco) and 100 μg/ml Penicillin/Streptomycin (P/S; Gibco) at 37 °C in a humidified incubator with 5% CO_2_.

### Mammalian lentiviral shRNA

For knockdown experiments, human EpCAM and HGFR shRNAs in the pLKO vector were obtained from the RNAi core facility at Academia Sinica. Lentivirus was produced according to standard protocols with minor modifications. In brief, 293T cells were seeded at a density of 70% in a 100-mm dish and transfected with packaging vectors (i.e., pCMV-ΔR8.91, containing gag, pol, and rev genes), envelope vectors (i.e., pMD2.G; VSV-G expressing plasmid), and an individual shRNA vector. The shRNA plasmids were transfected into 293T cells using poly-jet transfection reagent (SignaGen Laboratories). After an overnight incubation, the medium was changed to BSA-containing media. HCT116 cells were infected with viral supernatant containing polybrene (8 µg/ml) for 24 h. Then, the infection procedure was repeated, and cells were incubated in puromycin (2 μg/ml) for 7 days to select those with stable shRNA expression.

### EpCAM gene knockout

For the EpCAM knockout, CRISPR/cas9 gRNA constructs were purchased from Genescript. To produce the lentivirus, 293T cells were transiently transfected with CRISPR/cas9 gRNA plasmids, the EpCAM gRNA (target sequence: GTGCACCAACTGAAGTACAC), packaging plasmid (pCMV-ΔR8.91) and an envelope expression plasmid (pMD.G). HCT116 or HT29 cells were cultured with lentivirus-containing medium and selected with 2 μg/ml puromycin. Single cell clones were isolated from the selected pool, and the expression of EpCAM was examined with Western blotting.

### Production and purification of EpEX-His recombinant protein

The recombinant protein was expressed and purified using the Expi293 expression system. Cells were grown in Expi293 expression medium, and protein expression was induced by the addition of an enhancer reagent. The supernatant was harvested by centrifugation. After centrifugation at 8000×*g* for 20 min at 4 °C, the supernatant was incubated with nickel-chelated affinity resin (Ni–NTA, Qiagen) for 2 h at 4 °C. The resin was washed with wash buffer containing 50 mM Tris–HCl (pH 8.0), 500 mM NaCl, and 20 mM imidazole, and the proteins were eluted with elution buffer containing 50 mM Tris–HCl (pH 8.0), 500 mM NaCl, and 250 mM imidazole.

### Construction of the EpCAM EGF-like domain deletion mutant

In its extracellular domain, EpCAM contains two EGF-like domains at amino acids 27–59 (EGF-like domain I) and 66–135 (EGF-like domain II) and a cysteine-free motif [27]. The EpCAM EGF-like domain deletion mutant was generated using a standard QuikChange™ deletion mutation system with 1st forward mutagenic deletion primer (5′-GCAGCTCAGGAAGAATCAAAGCTGGCTGCC-3′), 1st reverse mutagenic deletion primer (5′-GGCAGCCAGCTTTGATTCTTCCTGAGCTGC-3′), 2nd forward primer (5′-AAGCTGGCTGCCAAATCTGAGCGAGTGAGA-3′) and 2nd reverse primer (5′-TCTCACTCGCTCAGATTTGGCAGCCAGCTT-3′). The PCR amplifications were performed using KAPA HiFi Hot Start DNA polymerase (Kapa Biosystems), and products were treated with a restriction enzyme, DpnI (Thermo Scientific), to digest methylated parental DNAs.

### Immunoprecipitation assay

Cells were lysed in lysis buffer (50 mM Tris–HCl, pH 7.4, 150 mM NaCl, and 1% NP-40) with Protease Inhibitors (Roche). For immunoprecipitation, cell lysates were incubated with antibodies for 6 h at 4 °C. Then, 20 μl Dynabeads Protein G was added, and the mixture was incubated for 2 h at 4 °C to pull-down the antibody-bound protein. The immunoprecipitation samples were washed with PBS three times, denatured in sample buffer, and analyzed by Western blotting.

### Generation of monoclonal antibodies and purification of IgG

EpAb2-6 and control IgG were generated as described previously [10]. The experimental protocol was approved by the Committee on the Ethics of Animal Experiments of Academia Sinica (AS IACUC: 11-04-166).

### Protein extraction and immunoblotting

Whole-cell extracts were prepared with RIPA buffer (50 mM Tris–HCl pH7.4, 1% NP-40, 0.5% Sodium deoxycholate, 0.1% SDS, 150 mM NaCl, 2 mM EDTA, 50 mM NaF). Protein concentrations of the cell lysates were determined by the Bradford assay. The lysates were separated on a 10% polyacrylamide gel and then transferred to the PVDF membrane. The membrane was blocked for 1 h with 3% BSA in PBST. The membrane was then incubated overnight with primary antibodies. Appropriate horseradish peroxidase-associated secondary antibodies (Millipore) were applied, and the membranes were incubated at room temperature (RT) for 1 h. The protein bands were subsequently visualized with chemiluminescence reagents (Millipore) and detected on a BioSpectrum 600 Imaging system (UVP). The protein level was quantified from band intensity using Gel-Pro analyzer 3.1 (Media Cybernetics).

### Cycloheximide chase assay

Cycloheximide, a protein synthesis inhibitor, was used to evaluate the stability of Snail. Cells were treated with cycloheximide for 0, 0.5, 1, or 2 h. Proteins were extracted, and Western blot analysis was performed to detect the Snail protein level.

### Cell viability assay

Cell viability was assayed by measuring mitochondrial dehydrogenase activity with the WST-1 (4-[3-(4-lodophenyl)-2-(4-nitrophenyl)-2*H*-5-tetrazolio-1, 3-benzene disulfonate) assay. Cells were seeded in 96-well plates at a density of 10^4^ cells/well and cultured for 24 h. New culture media with EGF, EpEX, or deglycan-EpEX at the indicated concentrations were added to the cells. At the end of the treatment period, 10 μl of WST-1 proliferation reagent (5 μg/ml) was added to each well and incubated for 1 h at 37 °C. Following the incubation, the absorbance of each well was detected at 450 nm using a spectrophotometric microplate reader.

### Total internal reflection fluorescence microscopy (TIRFM) and fluorescence resonance energy transfer (FRET)

Cells were cultured on the cover glass overnight. To observe the HGFR-EpEX interaction, the cells were fixed and costained with HGFR or EpEX antibodies as described earlier in the IFS with cell lines section. The imaging acquisition was performed in Single-molecule Biology Core Lab, Academia Sinica. Briefly, the TIRFM system was built on an inverted microscope (Olympus IX81). The system was equipped with a high-sensitivity EMCCD camera (iXOn3-897, Andor Technology) and a UPONAPO 100X OTIRF objective lens (NA:1.49; Olympus) to achieve TIRF. For the FERT event, the donor channels (HGFR-568 AF488) were excited with 488 solid lasers (50 mW, 10% power) and the signals from acceptor channel (EpEX-AF568) were detected with DV2. The Xcellence software (Olympus imaging software) was used to control the TIRFM.

### Colony formation assay

Cells were seeded in 24-well plates (1 × 10^4^ cells/well) and cultured for 7 days. The cells were then fixed with 4% formaldehyde and stained with crystal violet. After capturing images of the plates, a solution with 0.5% SDS was added to each well, and the plates were incubated for 2 h at room temperature. The relative densities of cells were then determined by measuring the absorbance of the solution at 570 nm using a microplate reader. The experiments were performed in triplicate.

### Transwell migration and invasion assay

Cell migration and invasion were assayed with 8-μm pore size Transwell migration chambers (Millicell) without or with 10% matrigel. Cells (1 × 10^5^) were added to the upper chamber in 500 μl serum-free DMEM. Then, as a chemoattractant, 700 μl DMEM containing 10% FBS was added to the lower chamber. Migration and invasion were allowed to proceed for 16 h at 37 °C in a standard cell culture incubator. Then, cells were removed from the upper surface of the membranes with cotton swabs, and the cells migrated to the lower surface were stained with 0.05% (w/v) crystal violet in 4% paraformaldehyde (in 1× PBS) for 15 min and washed with water. Membranes were dried for 15–20 min before at least four random fields on the membrane examined at high power were counted for each experimental condition.

### Apoptosis assays

Cells were seeded and treated with 10 μg/ml mAb or inhibitor for 6 h; an unrelated mouse myeloma immunoglobulin used at appropriate dilution served as the IgG2a (Invitrogen #02-6200) isotype control. Apoptotic cells were detected using Annexin V-FITC and PI and were analyzed using a flow cytometer (BD Immmunocytometry Systems). Early apoptosis was measured with the Annexin V-FITC Apoptosis Detection kit II (BD Pharmingen). Late apoptotic nuclei were detected with propidium iodide (PI) staining.

### RNA extraction, cDNA synthesis, quantitative reverse transcription polymerase chain reaction (qRT-PCR)

Total RNA extraction, first-strand cDNA synthesis, and SYBR-green-based real-time PCR were performed as described in the manufacturer's instructions. To extract total RNA, cells were lysed using TRIzol reagent (Invitrogen), and proteins and phenol were removed from TRIzol using chloroform. After centrifugation, the top colorless layer was collected and mixed with isopropanol to precipitate the RNA pellet. The RNA pellet was then washed with 70% ethanol, air-dried at room temperature, and dissolved in RNase-free water. For first strand cDNA synthesis, 5 μg of total RNA was used for reverse transcription with oligo (dT) primer and SuperScriptIII reverse transcriptase (Invitrogen) at 50 °C for 60 min. Target gene levels were evaluated by quantitative PCR (qPCR), using LightCycler 480 SYBR Green I Master Mix (Roche) and a LightCycler480 System (Roche). GAPDH mRNA expression was measured as endogenous housekeeping control to normalize all q-PCR reactions. The qPCR reaction was 95 °C for 5 min, followed by 40 cycles of denaturation at 95 °C for 10 s, annealing at 60 °C for 10 s and extension at 72 °C for 30 s. The final results were calculated from three independent experiments. Primer sequences used to detect the mRNA expression of genes of interest are listed in Additional file 1: Table S1.

## Colon cancer metastatic animal models

Colon cancer cells (5 × 10^6^ cells/mouse) in PBS were injected into 4–6-week-old female NOD/SCID mice through the tail vein. Mice were then randomly assigned to different treatment groups by body weight. After 3 days, antibodies were administered through tail vein injection twice a week for 4 consecutive weeks. In addition, Crizotinib was administered daily by oral gavage for 5 days per week (treatment for 4 weeks). For the therapeutic study, tumor-bearing mice were treated with isotype control IgG1 (15 mg/kg), crizotinib (20 mg/kg), EpAb2-6 (15 mg/kg), or crizotinib (20 mg/kg) combined with EpAb2-6 (15 mg/kg). Mouse survival rate were measured. Animal care was carried out in accordance with the guidelines of Academia Sinica, Taiwan. The protocol was approved by the Committee on the Ethics of Animal Experiments of Academia Sinica (AS IACUC: 20-05-1468).

### Orthotopic implantation and therapeutic studies

Orthotopic tumor models were created as previously reported [9]. Briefly, NSCID mice were used for orthotopic implantation of colon cancer cells previously infected with Lenti-Luc virus (lentivirus containing luciferase gene). The mice were anesthetized by i.p. injection of Avertin, 2,2,2-Tribromo-ethanol (Sigma-Aldrich) at 250 mg/kg. Tumor development was monitored by bioluminescence imaging. For the therapeutic study, tumor-bearing mice were treated with isotype control IgG1 (15 mg/kg), crizotinib (20 mg/kg), EpAb2-6 (15 mg/kg), or crizotinib (20 mg/kg) combined with EpAb2-6 (15 mg/kg). Tumor progression was monitored by quantification of bioluminescence. Mouse survival was also monitored. Animal care was carried out in accordance with the guidelines of Academia Sinica. The protocol was approved by the Committee on the Ethics of Animal Experiments of Academia Sinica (AS IACUC: 20-05-1468).

### Statistical analysis

All data are presented as mean ± SEM for the indicated number of experiments. Unpaired Student’s *t*-test was used to analyze the expression percentages in experimental versus control cultures. A *p*-value of less than 0.05 was considered statistically significant.

## Results

### EpEX interacts with HGFR and induces HGFR phosphorylation

**Fig. 1:**
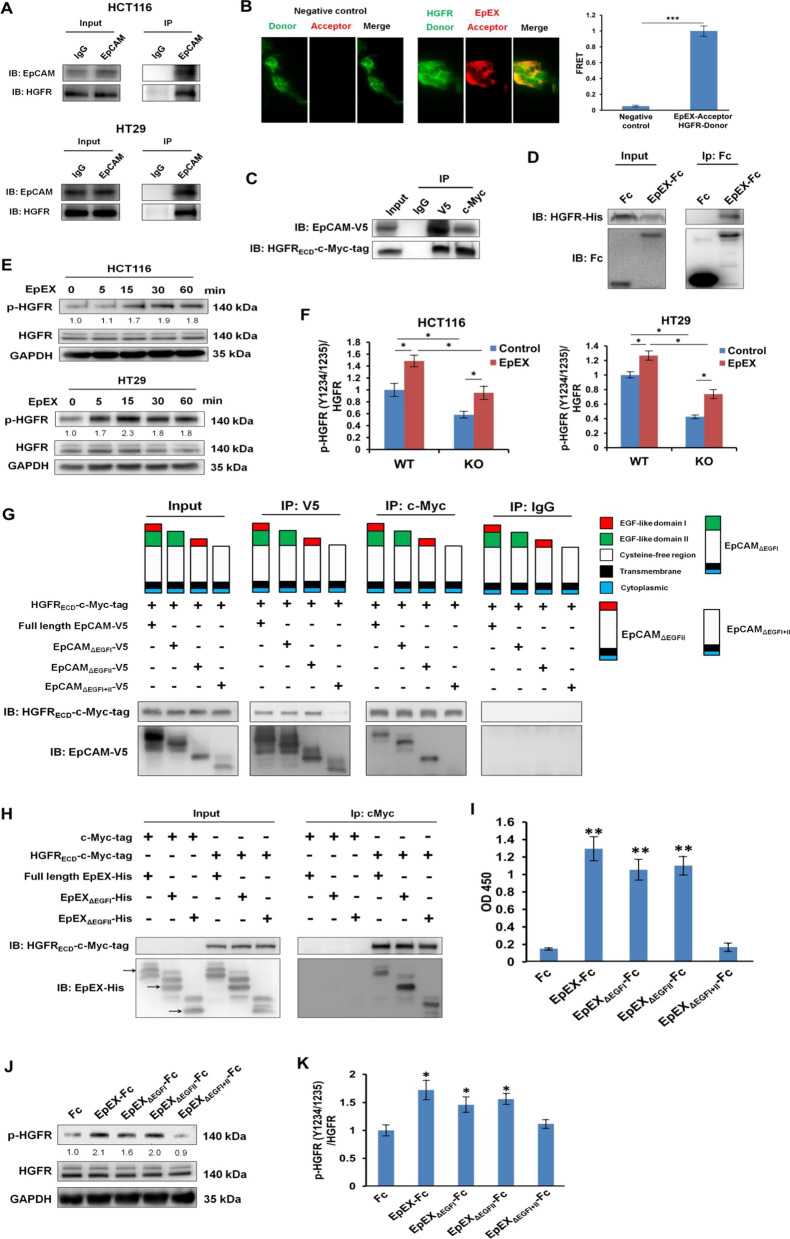
EpEX interacts with HGFR and induces HGFR phosphorylation. **A** Immunoprecipitation (IP) of endogenous EpCAM bound to HGFR in HCT116 and HT29 cells. **B** Representative images of TIRF-FRET experiments showing energy transfer from HGFR to EpEX in HCT116 cells. **C** HEK293T cells were transfected with HGFR_ECD_-c-Myc and EpCAM-V5. IP was performed with control IgG, anti-V5 antibody, or anti-c-Myc antibody followed by Western blotting. **D** EpEX-Fc and HGFR-His recombinant protein (2.5 μg/ml) interaction were examined by IP with Dynabeads Protein G and Western blotting with anti-6× His tag antibody. **E** Starved HCT116 and HT29 cells were treated with 50 nM EpEX-His for the indicated times. The phosphorylation of HGFR was examined by Western blotting. **F** Wild-type (WT) or EpCAM knockout (KO) HCT116 and H29 cells were starved for 16 h and then treated with EpEX-His (50 nM) for 15 min. The level of phosphorylated HGFR was assayed with an ELISA kit (ab126451). **G** HEK293T cells were transfected with HGFR_ECD_-c-Myc and full-length or EGF-like-domain deletion mutant EpCAM-V5. The protein interaction was probed by IP with anti-V5 or anti-c-Myc antibodies and Western blotting with anti-V5 or anti-cMyc antibodies. **H** HEK293T cells were transfected with c-Myc or HGFR_ECD_-c-Myc and full-length or EGF-like-domain deletion mutant EpEX-His. The protein interaction was probed by IP with anti-c-Myc antibodies and Western blotting with anti-cMyc and anti-His antibodies. **I** HGFR-His recombinant protein (2 μg/ml) was added to EGF-like-domain deletion mutant-Fc-coated (1 μg/ml) ELISA plates and detected by TMB colorimetric peroxidase assay. HCT116 cells were starved and treated with wild-type or EGF-domain deletion mutant EpEX, and phosphorylated HGFR was analyzed by **J** Western blotting and **K** ELISA kit (ab126451). Statistical differences were determined by two-tailed Student *t-test*. *N* = 3 independent experiments. All data are presented as mean ± SEM. *p < 0.05; **p < 0.01

In our previous study, we conducted a Human Phospho-RTK Array Kit (R&D Systems) assay and found that EpEX induces both EGFR and HGFR phosphorylation in HCT116 cells [5]. To test whether endogenous EpCAM directly interacts with HGFR in HCT116 and HT29 colon cancer cell lines, we used DTSSP, a cross-linker, to stabilize the putative EpCAM-HGFR complex. As we predicted, the interaction of EpCAM and HGFR was confirmed by immunoprecipitation (IP) and Western blotting (Fig. 1A). To further study whether the membrane-bound EpCAM could bind to the extracellular domain of HGFR (HGFR_ECD_), we performed co-IP experiments using HEK293T cells that overexpress both EpCAM-V5 and HGFR_ECD_-c-Myc-tag. In order to further confirm that EpEX binds to HGFR, we performed a direct binding assay using total internal reflection fluorescence (TIRF) microscopy combined with fluorescence resonance energy transfer (FRET) techniques. Using HGFR as the donor channel (AF488), we could successfully observe energy transfer to the EpEX (AF568) used as the acceptor channel, suggesting EpEX binding to HGFR (Fig. 1B). The results confirmed interactions between exogenous EpCAM and HGFR (Fig. 1C). Next, we performed IP to probe the direct interaction between recombinant EpEX-Fc and HGFR_ECD_-His recombinant protein (Fig. 1D). To investigate the effect of EpEX on the phosphorylation of HGFR in colon cancer cells, we analyzed the levels of phosphorylated HGFR in HCT116 and HT29 cells. The Western blotting and ELISA results showed that EpEX induced phosphorylation of HGFR in both cell types. In addition, EpEX could induce HGFR phosphorylation in the absence of EpCAM (Fig. 1E, F).

EpEX comprises two EGF-like domains [27]; hence we sought to determine which domain interacts with HGFR. To do so, we constructed various EGF-like domain deletion mutant (EpCAM_ΔEGFI_, EpCAM_ΔEGFII_, and EpCAM_ΔEGFI+II_) plasmids. Surprisingly, the mutants harboring only one EGF-like domain deletion (EpCAM_ΔEGFI_ and EpCAM_ΔEGFII_) could interact with HGFR. In contrast, mutants with both domains deleted mutants (EpCAM_ΔEGFI+II_) could not interact with the receptor (Fig. 1G). A similar result was observed when assessing HGFR_ECD_ binding with soluble EpEX wild-type or mutant proteins (Fig. 1H). Overall, these findings indicate that membrane-bound EpCAM and secreted EpEX can both bind HGFR through either EGF-like domain I or II of EpCAM/EpEX.

Next, we performed ELISA to probe the potential interactions between several variants of EGF-like-domain-deleted mutants of EpEX-Fc and HGFR-His proteins. The results confirmed EpEX could bind HGFR via either domain, but HGFR binding to EpEX_ΔEGFI+II_ mutant protein was abolished entirely (Fig. 1I). Similar to previous phosphorylation results on wild-type EpEX (Fig. 1D, E), we observed both EpEX_ΔEGFI_ and EpEX_ΔEGFII_ could induce HGFR phosphorylation; however, EpEX_ΔEGFI+II_ protein could not (Fig. 1J, K). Based on these results, we conclude that both EGF-like domains of EpEX may bind to HGFR and induce consequent phosphorylation.

### EpEX induces HGFR signaling and promotes cancer cell growth

**Fig. 2:**
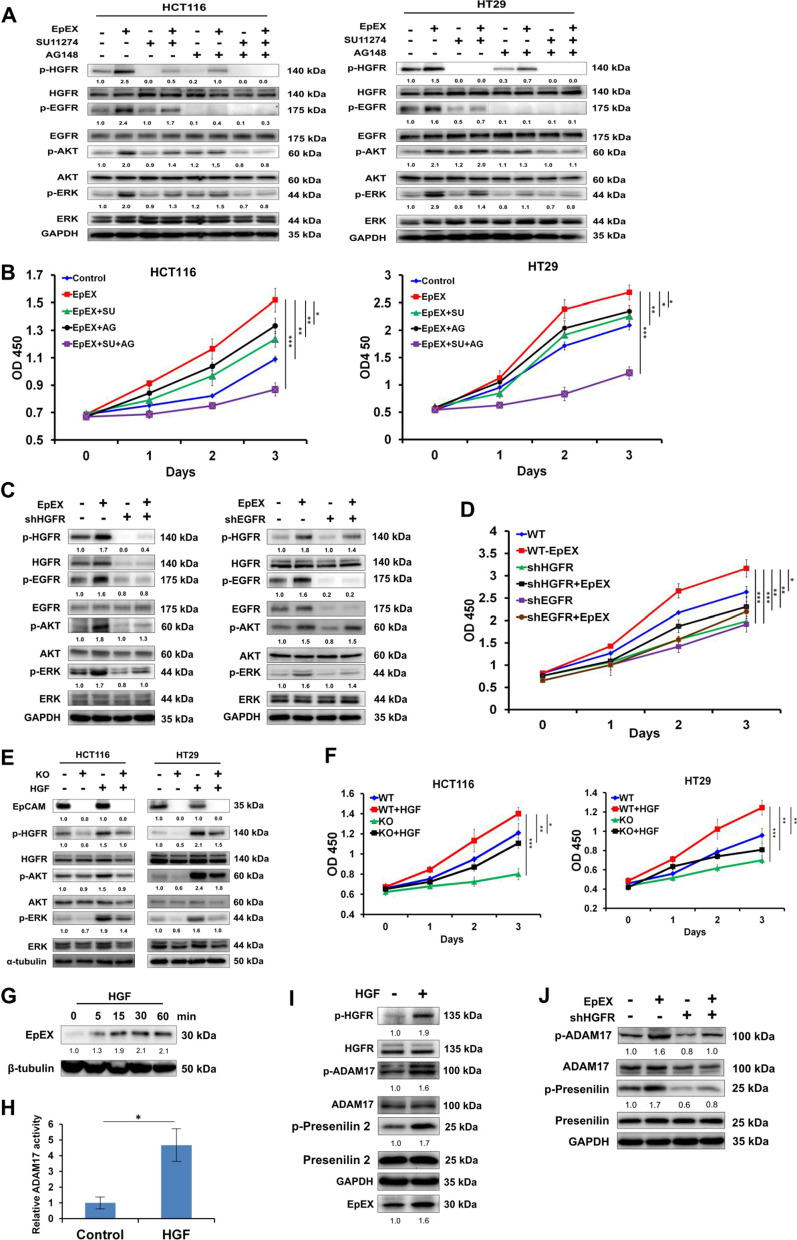
EpEX induces cancer growth via HGFR signaling pathway. **A** Starved HCT116 and HT29 cells were treated with HGFR inhibitor SU11274 (SU, 10 μM) and EGFR inhibitor AG1478 (AG, 10 μM) for 1 h, then treated with 50 nM of EpEX-His for 15 min. The levels of phosphorylated HGFR, EGFR, AKT, and ERK were examined by Western blotting. **B** HCT116 and HT29 cells were treated with 50 nM EpEX, SU (10 μM), and AG (10 μM). Cell growth was examined by WST-1 assay after treatment for the indicated time. **C** HCT116 cells were treated with Luc, HGFR, or EGFR shRNA and then treated with 50 nM of EpEX-His for 15 min. The levels of phosphorylated HGFR, EGFR, AKT, and ERK were examined in HGFR knockdown HCT116 cells by Western blotting. **D** HCT116 cells were treated with Luc, HGFR, or EGFR shRNA and then treated with 50 nM of EpEX-His for indicated times. Cell growth was examined by the WST-1 assay. **E** WT or EpCAM knockout (KO) HCT116 and HT29 cells treated with HGF (0.5 nM) for 15 min. The phosphorylation of HGFR, AKT, and ERK was examined by Western blotting. **F** WT or KO HCT116 and HT29 cells were treated with HGF (0.5 nM) with 2% FBS for the indicated times. Cell growth was examined by the WST-1 assay. **G** HCT116 cells were treated with HGF (0.5 nM) for indicated times, and the EpEX protein level in culture medium was examined by immunoprecipitation and Western blotting. **H** HCT116 was treated with 0.5 nM HGF for 15 min, and ADAM17 activity was measured. **I** HCT116 cells were treated with HGF (0.5 nM) for 15 min. The phosphorylated HGFR, presenilin 2, and ADAM17 protein level in cell lysates were analyzed by Western blotting, and the EpEX protein level in culture medium was examined by immunoprecipitation and Western blotting. **J** HCT116 cells were treated with shLuc or HGFR shRNA. Then, the cells were treated with 50 nM EpEX-His for 15 min. The levels of phosphorylated ADAM17 and presenilin 2 were examined by Western blotting. Statistical differences were determined by two-tailed Student *t* test. *N* = 3 independent experiments. All data are presented as mean ± SEM. *p < 0.05; **p < 0.01; ***p < 0.001

The ability of EpCAM to induce HGFR phosphorylation suggested that this pathway might be partially responsible for tumorigenicity in colon cancer cells. Previous studies indicated that HGFR can crosstalk to EGFR signaling. We found that the HGFR inhibitor (SU11274) more significantly attenuates EpEX-mediated ERK and AKT phosphorylation than the EGFR inhibitor (AG1478). Furthermore, the combination of these two inhibitors reduced ERK and AKT phosphorylation levels compared to controls and single treatment conditions (Fig. 2A). We then wanted to further understand the effects of such inhibition on cell growth. We found that SU11274 and AG1478 abolished the EpEX-induced increases in cell growth in colon cancer cells (Fig. 2B).

We noticed that HGFR knockdown significantly reduces EpEX-induced phosphorylation of EGFR, AKT, and ERK, while EGFR knockdown only significantly reduces EpEX-induced ERK phosphorylation (Fig. 2C). In addition, EpEX-induced cell growth was significantly reduced by HGFR or EGFR knockdown in HCT116 cells (Fig. 2D).

To investigate whether EpCAM and HGFR activation cooperatively regulates cancer progression and metastasis, we examined the phosphorylated ERK and AKT levels in EpCAM knockout cells with or without HGF treatment. Western blotting showed that the levels of phosphorylated HGFR, AKT and ERK were significantly decreased in HGF-treated EpCAM knockout cells compared to those in HGF-treated wild-type cells (Fig. 2E). Further, results of the cell growth assay showed that EpCAM knockout cells grew slower than wild-type HCT116 and HT29 cells, but the trajectory of cell growth could be restored by treating the EpCAM knockout HCT116 and HT29 cells with HGF (Fig. 2F).

We also found that EpEX production was elevated after HGF treatment of HCT116 cells using an IP assay (Fig. 2G). Since EpCAM was cleaved by active-ADAM17 (TACE) to release soluble EpEX, the results indicated that HGF treatment could induce ADAM17 activity in HCT116 cells (Fig. 2H). This observation was in line with our further finding that HGF treatment increased RIP (phosphorylated ADAM17 and presenilin 2) and subsequent EpEX production in HCT116 cells (Fig. 2I). Moreover, HGFR knockdown could diminish EpEX-induced phosphorylation of ADAM17 and presenilin 2 (Fig. 2J).

### EpEX activates ERK and FAK-AKT signaling

**Fig. 3:**
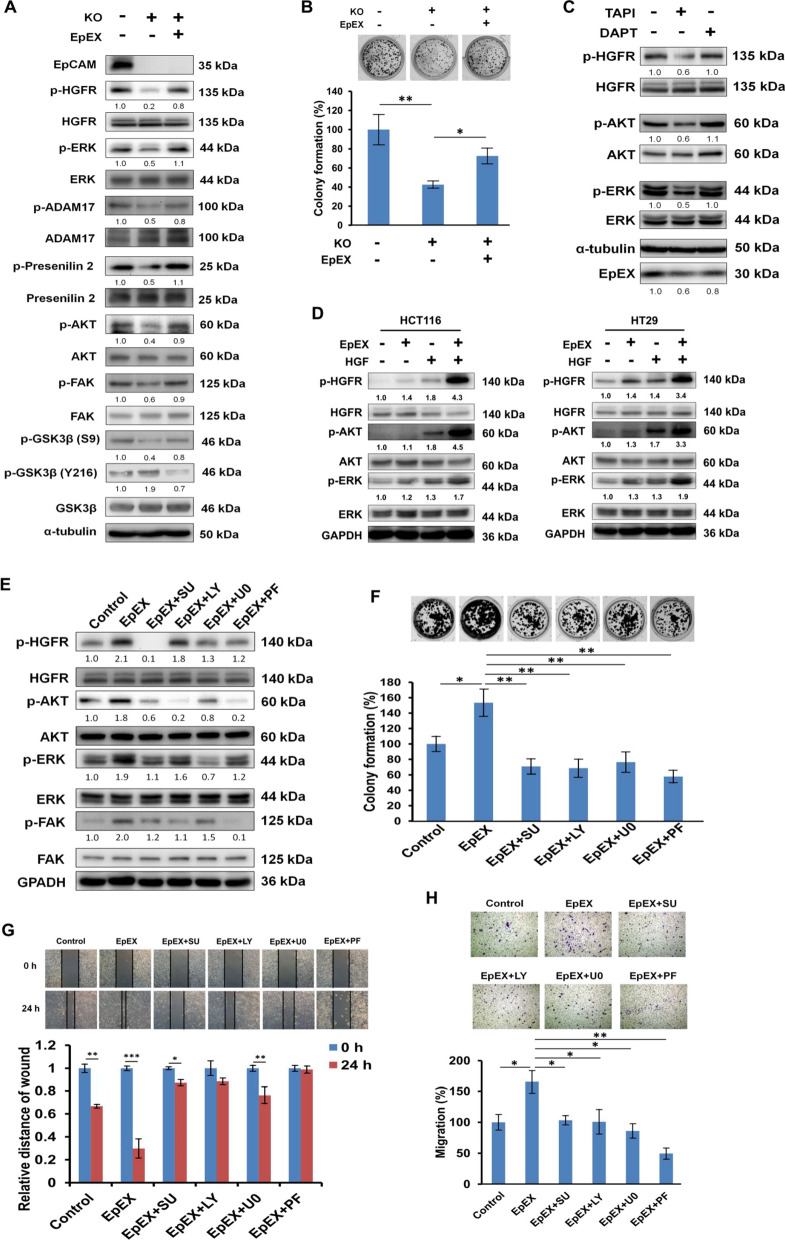
EpEX induces the activation of ERK and FAK-AKT signaling pathway. Wild type (WT) or EpCAM knockout (KO) HCT116 cells after EpEX-His treatment. **A** The levels of phosphorylated HGFR, AKT, FAK, GSK3β, ERK, ADAM17, and presenilin 2 were assayed by Western blotting. **B** Colony formation was examined by crystal violet staining. **C** HCT116 cells were treated with TAPI (ADAM17 inhibitor) or DAPT (γ-secretase inhibitor) for 24 h, and the phosphorylation of HGFR, AKT, and ERK was analyzed by Western blotting, and EpEX protein level in culture medium was examined by immunoprecipitation and Western blotting. **D** HCT116 and HT29 cells were starved for 16 h, then treated with EpEX-His (50 nM) and HGF (0.5 nM) for 15 min. The levels of phosphorylated HGFR, AKT, and ERK were examined by Western blotting. **E** HCT116 cells were starved for 16 h, then treated with 50 nM EpEX-His for 15 min. HGFR inhibitor SU11274 (SU, 10 μM), AKT inhibitor LY294002 (LY, 25 μM), ERK inhibitor U0126 (U0, 20 μM), or FAK inhibitor PF-562271 (PF, 10 μM) were applied 1 h before EpEX treatment. Phosphorylation of AKT, ERK, and FAK was examined by Western blotting. **F** HCT116 cells were pretreated with SU (10 μM), LY (25 μM), U0 (20 μM) or PF (10 μM) for 1 h and then treated with 50 nM EpEX. The treatments were changed every 3 days along with the medium. Colony formation was examined by crystal violet staining after 7 days. The relative colony densities are shown. **G** Migration ability was examined by the wound healing assay at the indicated times. **H** The numbers of migration cell was assessed by a Transwell after treatment for 24 h. Statistical differences were determined by two-tailed Student *t* test. *N* = 3 independent experiments. All data are presented as mean ± SEM. *p < 0.05; **p < 0.01

EpCAM is known to influence the growth, survival, and metastasis of cancer cells via its downstream effectors. In its signaling process, the proteolysis of EpCAM produces EpEX, which may further stimulate RIP and the release of EpICD that subsequently transduces EpCAM signaling [4]. Moreover, it was previously shown that EpEX treatment to HCT116 cells could increase RIP via phosphorylation of TACE and presenilin 2, the catalytic subunit of γ-secretase [5]. We, therefore, treated EpCAM knockout HCT116 cells with EpEX; the treatment resulted in partial restoration of HGFR downstream signaling, including phosphorylation of AKT, FAK, and ERK as well as phosphorylation of RIP proteins (ADAM17 and presenilin 2) (Fig. 3A). Previous work showed that GSK3β antagonists stimulate EMT via AKT [28]. In line with this mechanism, we found that EpEX could rescue suppressive phosphorylation of GSK3β (S9, inactive GSK3β) while it simultaneously decreased activating phosphorylation of GSK3β (Y216, active GSK3β) in EpCAM knockout cells (Fig. 3A). This result was confirmed in HT29 cells, as the levels of phosphorylated HGFR, AKT and ERK proteins were all partially rescued in EpCAM knockout HT29 cells after treatment with EpEX (Additional file 1: Fig. S1A). We also found that EpEX increased colony formation in EpCAM knockout HCT116 cells (Fig. 3B), and blocking the shedding of endogenous EpEX (but not EpICD) decreased HGFR, AKT, and ERK phosphorylation. These results suggested that endogenous EpEX may be crucial for HGFR signaling activation (Fig. 3C).

We next tested whether EpEX could enhance HGF-induced HGFR signaling. Incubation of HCT116 and HT29 cells with soluble EpEX in combination with HGF upregulated phosphorylation of HGFR and subsequent downstream, including AKT and ERK, compared to EpEX or HGF treatments alone (Fig. 3D). Since EpEX can trigger HGFR activation, we further investigated the effects of EpEX on the mediators of HGFR signaling. In this context, AKT and ERK signaling are two of the most important cancer-associated signaling pathways [29, 30], as the pathways play a variety of physiological roles in regulating EMT, cell cycle, survival, and cancer progression. Therefore, we tested whether an HGFR inhibitor (SU11274), AKT inhibitor (LY294002), ERK inhibitor (U0126), and FAK inhibitor (PF-562271) could affect EpEX-induced signaling in HCT116 cells. We noted that EpEX increased the AKT, ERK, and FAK phosphorylation levels (Fig. 3E), colony formation potentials (Fig. 3F), wound healing (Fig. 3G), and migration (Fig. 3H) abilities. Together, these results indicate that EpEX increases ERK and FAK-AKT signaling pathways by inducing HGFR activation in colon cancer cells.

### EpEX promotes EMT and invasion by inducing active β-catenin and Snail expression via down-regulating GSK3β activity

We used shRNA to silence EpCAM expression in HCT116 and HT29 cells; the results showed that knockdown of EpCAM significantly diminished cell migration and invasion (Additional file 1: Fig. S2A). Furthermore, we found that EpCAM knockdown in HCT116 and HT29 cells increased E-cadherin expression and reduced Snail's expression (Additional file 1: Fig. S2B).

**Fig. 4:**
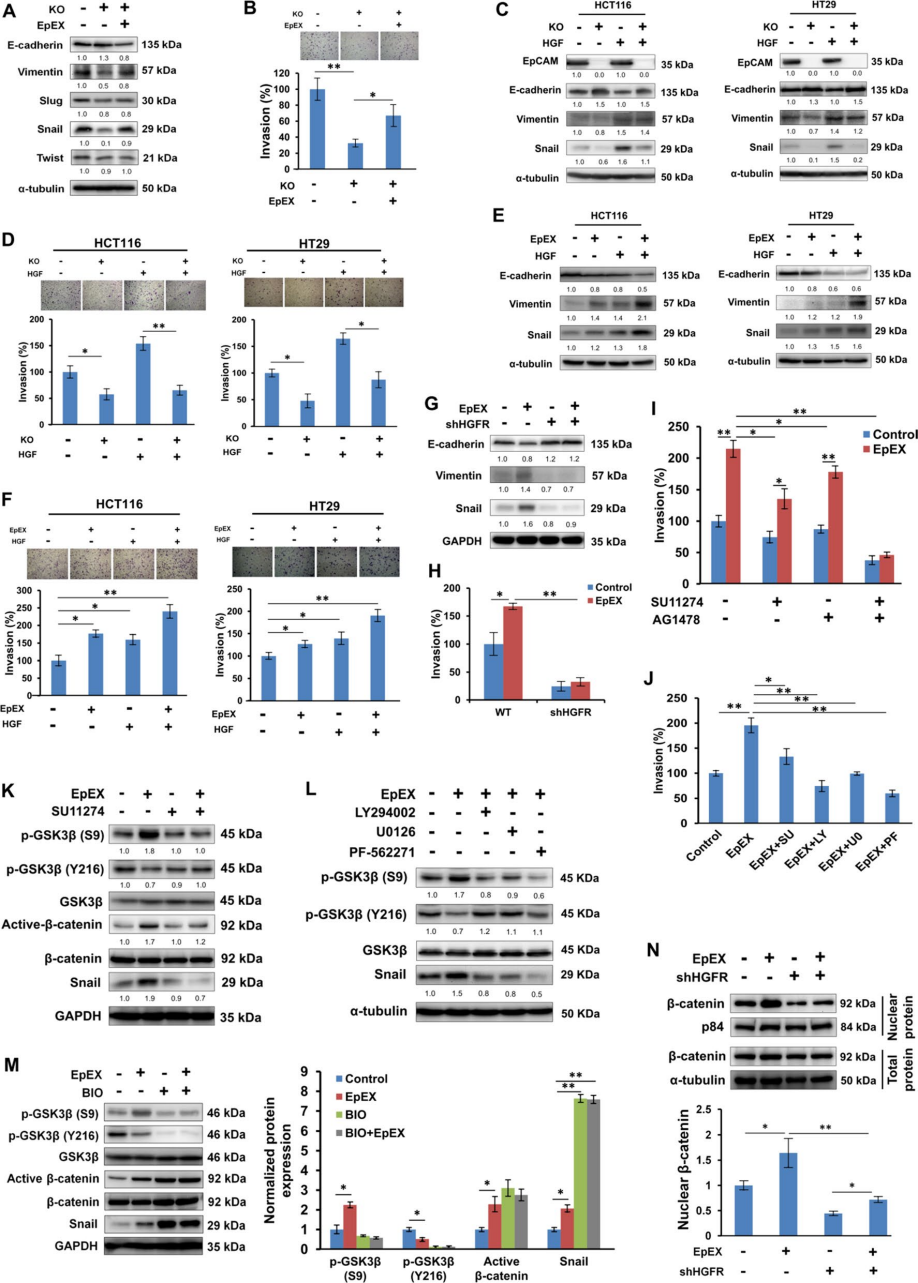
EpEX induces EMT and invasion by stabilizing β-catenin and Snail through decreasing GSK3β activity. **A** The protein expression of EMT markers and regulators was detected by Western blotting in Wild-type (WT) or EpCAM knockout (KO) HCT116 cells after EpEX treatment. **B** Cell invasion was examined by Transwell chamber assay with matrigel. **C** WT or KO HCT116 and HT29 cells were starved for 16 h, then treated with 0.5 nM of HGF with 2% FBS for 24 h. EMT-related protein expression (E-cadherin, vimentin, and Snail) was examined by Western blotting. **D** WT or KO HCT116 and HT29 cells were treated with HGF (0.5 nM) for 24 h. Invasion by HCT116 and HT29 cells was examined by Transwell chamber assay with matrigel. **E** HCT116 and HT29 cells were starved for 16 h, then treated with 50 nM of EpEX and 0.5 nM of HGF with 2% FBS for 24 h. EMT-related protein expression (E-cadherin, Vimentin, and Snail) was examined by Western blotting. **F** HCT116 and HT29 cells were starved for 16 h, then treated with 0.5 nM of HGF with 2% FBS for 24 h. Cell invasion was assessed by a Transwell assay with matrigel. **G** HCT116 cells after shLuc and shHGFR treatment were treated with 50 nM of EpEX-His. HCT116 cells after shLuc and shHGFR treatment were treated with 50 nM of EpEX-His with 2% FBS for 24 h. EMT-related protein expression (E-cadherin, Vimentin, and Snail) was examined by Western blotting. **H** Cell invasion was examined by Transwell chamber assay with matrigel. **I** HCT116 cells were treated with HGFR inhibitor SU11274 (10 μM) and EGFR inhibitor AG1478 (10 μM) for 1 h then treated with 50 nM of EpEX-His for 24 h. Cell invasion was examined by Transwell chamber assay with matrigel. **J** HCT116 cells were treated with SU (10 μM), LY (25 μM), U0 (20 μM) or PF (10 μM) for 1 h then treated with 50 nM of EpEX-His for 24 h. Cell invasion was examined by Transwell chamber assay with matrigel. **K** Starved HCT116 cells were treated SU (10 μM) for 1 h, followed by treatment with EpEX-His (50 nM) for 24 h. Phosphorylated GSK3β, active-β-catenin, and Snail were detected by Western blotting. **L** HCT116 cells were starved for 16 h, then treated with 50 nM EpEX-His for 24 h. AKT inhibitor LY294002 (25 μM), ERK inhibitor U0126 (20 μM), or PF-562271 (10 μM) were applied 1 h before EpEX treatment. Protein expression of phosphorylated GSK3β and Snail was examined by Western blotting. **M** Protein expression was analyzed by Western blotting in EpEX-His (50 nM) treated HCT116 cells after treatment with or without 2 μM GSK3β inhibitor (BIO) for 24 h. Quantification of the normalized protein expression in the right panel. **N** HCT116 cells were treated with shLuc or HGFR shRNA then treated with 50 nM EpEX-His for 24 h. Active β-catenin nuclear translocation was assayed by Western blotting. Quantification of the normalized protein expression in the lower panel. Statistical differences were determined by two-tailed Student *t* test. *N* = 3 independent experiments. All data are presented as mean ± SEM. *p < 0.05; **p < 0.01

Next, we found that EpCAM knockout inhibited the expressions of the mesenchymal marker vimentin, as well as the protein level of the EMT regulator Snail, while enhancing E-cadherin expression in HCT116 cells; of note, the EMT indicators were restored after EpEX treatment (Fig. 4A). EpEX also induced cell invasion in EpCAM knockout HCT116 (Fig. 4B) and HT29 (Additional file 1: Fig. S1B) cells.

To investigate whether EpCAM and HGFR activation cooperatively regulates cancer cell invasion, we examined EpCAM knockout cells with or without HGF treatment. We found that the expression of EMT-related proteins and cell invasion properties in EpCAM knockout HCT116 and HT29 cells were significantly reduced compared to wild-type cells. HGF-induced EMT and invasion activity were also reduced in EpCAM knockout cells (Fig. 4C, D). Incubation of HCT116 and HT29 cells with EpEX in combination with HGF upregulated the levels of EMT and cell invasion compared to EpEX or HGF treatments alone (Fig. 4E, F). Furthermore, EpEX-induced EMT-related protein expressions and cell invasion were prevented by HGFR knockdown (Fig. 4G, H). In contrast, EpEX-induced EMT and invasion were no significant inhibition by EGFR knockdown (Additional file 1: Fig. S3A, B). We also found that the HGFR inhibitors significantly attenuate EpEX-mediated phosphorylation of AKT and ERK and cell invasion (Additional file 1: Fig. S4A, B). The effects on EpEX-induced migration and invasion were even more strongly attenuated by the combination of HGFR inhibitor and EGFR inhibitor, compared to the treatment of HCT116 cells with single agents (Fig. 4I). EpEX-induced invasion was also suppressed by LY294002, U0126, and PF-562271 (Fig. 4J). These results suggest that EpEX can enhance HGFR activation and induce EMT and invasion in colon cancer cells.

Previous reports indicated that GSK3β antagonists stimulate EMT by AKT signaling, thus affecting Snail protein turnover via its phosphorylation and ubiquitin-mediated proteolysis [28]. In line with this mechanism, we found that EpEX could induce suppressive phosphorylation of GSK3β (S9, inactive GSK3β) while it simultaneously decreased activating phosphorylation of the protein (Y216, active GSK3β); these changes were coincident with increased Snail protein expression in HCT116 cells. However, SU11274 could attenuate EpEX-mediated GSK3β activity and abolish EpEX-induced active β-catenin and Snail protein expression in HCT116 cells (Fig. 4K). We also found that inhibitors (i.e., LY294002, U0126 or PF-562271) of HGFR downstream mediators could attenuate EpEX-mediated GSK3β activity and Snail protein expression (Fig. 4L). Of note, active β-catenin and Snail protein expression levels were upregulated both in control and EpEX-treated cells after treatment with the GSK3β inhibitor, BIO (Fig. 4M). We further confirmed that EpEX induced nuclear translation of active β-catenin via HGFR (Fig. 4N). These results suggest that EpEX promotes EMT and invasion by inducing active β-catenin and Snail protein expression via down-regulation of GSK3β activity.

### EpEX promotes Snail protein stability through the inhibition of ubiquitination-mediated proteasomal degradation

**Fig. 5:**
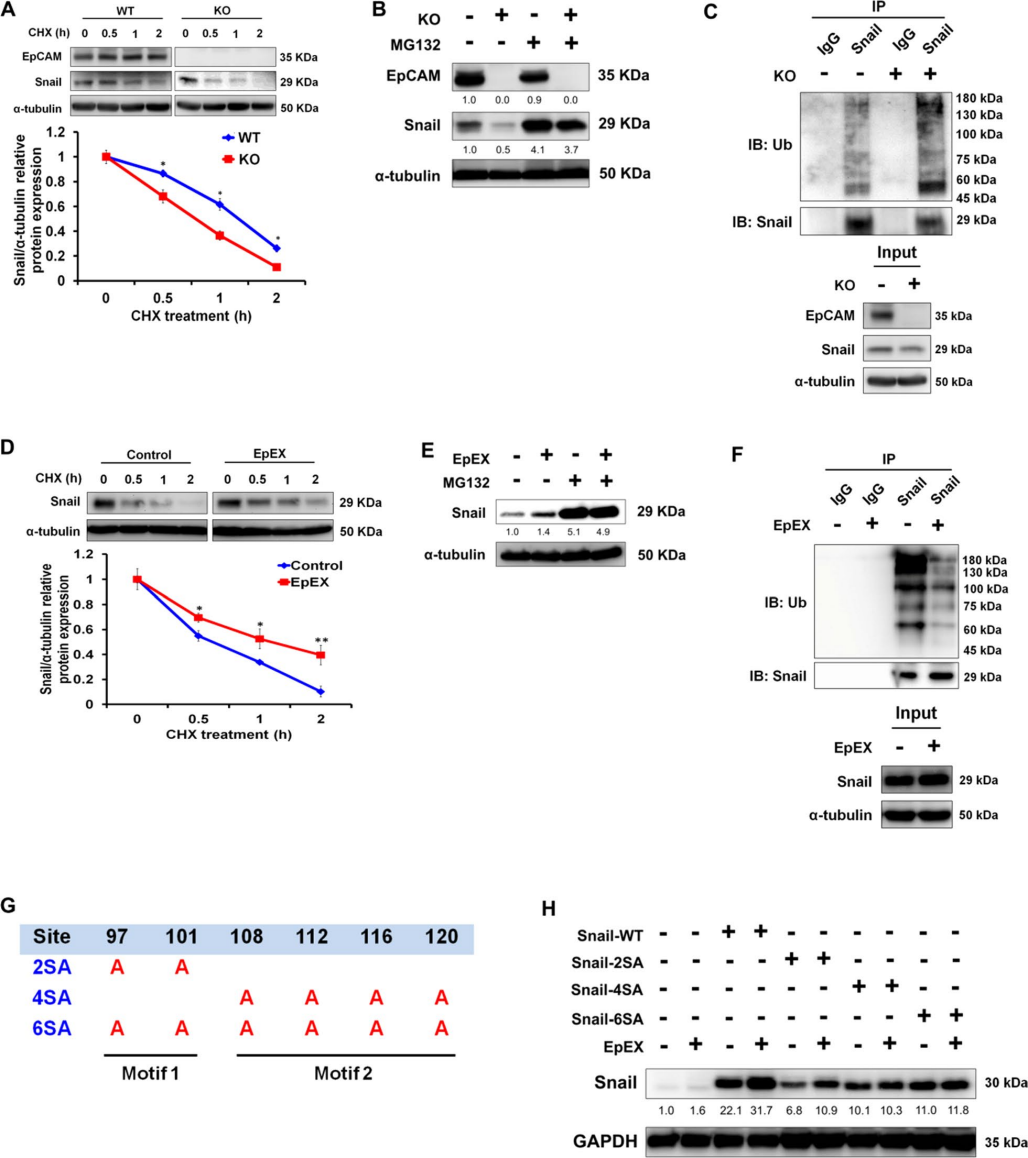
EpEX promotes Snail protein stability through inhibition of ubiquitination-mediated proteasomal degradation. (**A**) Stability of Snail protein in WT or KO of HCT116 cells. Cells were treated with cyclohexamide (CHX) 100 μg/ml at the indicated intervals and subjected to Western blotting. (**B**) The protein expression of Snail was analyzed in WT or KO HCT116 cells with or without 10 mM MG132 (proteasome inhibitor) treatment for 6 h, followed by Western blotting. (**C**) WT or KO HCT116 cells were treated with MG132 (10 μM) for 6 h before cell collection. The lysates were subjected to immunoprecipitation using anti-Snail antibody and input. Western blotting was performed with the indicated antibodies to detect ubiquitinated Snail protein. (**D**) Stability of Snail protein in HCT116 cells after EpEX (50 nM) treatment for 24 h. Cells were treated with cyclohexamide (CHX; 200 μg/ml) for the indicated intervals and then subjected to Western blotting. (**E**) Snail protein expression in HCT116 cells was analyzed by Western blotting after EpEX (50 nM) treatment for 24 h, and with or without 10 μM MG132 (proteasome inhibitor) treatment for 6 h. (**F**) HCT116 cells were treated with EpEX (50 nM) and MG132 (10 μM) for 6 h before cell collection. The lysates were subjected to immunoprecipitation using anti-Snail antibody and input. Western blotting was performed with the indicated antibodies to detect ubiquitinated Snail protein. (**G**) Schematic representation of positions of mutant within Snail phosphorylation motifs. (**H**) HCT116 cells were transfected with Snail-WT, 2SA, 4SA, and 6SA for 24 h and then further treated with or without EpEX (50 nM) for 24 h. The expression of Snail was detected by Western blotting. Statistical differences were determined by two-tailed Student *t* test. *N* = 3 independent experiments. All data are presented as mean ± SEM. *p < 0.05; **p < 0.01; ***p < 0.001

Expression and stabilization of Snail protein is involved in EMT and cancer metastasis [31], so we wanted to determine if EpCAM regulates Snail expression. We found that EpEX increased the protein expression level of Snail (Fig. 4A). Intriguingly, EpCAM knockout did not affect *SNAIL* gene expression, but it did lead to upregulation of *CDH1 (E-cadherin)* expression and downregulation of *VIM* and *SLUG* expression (Additional file 1: Fig. S5). These results suggested the possible involvement of EpCAM in stabilization of Snail protein at a post-translational level. Therefore, we treated the cells with cycloheximide that revealed the half-life of Snail protein was shortened upon knockout of EpCAM (Fig. 5A). On the other hand, treatment with MG132 (an inhibitor of the 26S proteasome) increased Snail steady-state protein levels, suggesting the protein level is controlled mainly by proteasomal degradation (Fig. 5B). EpCAM knockout also led to an increased level of ubiquitylated Snail compared to that of the control cells (Fig. 5C). Moreover, cycloheximide treatment further confirmed that EpEX treatment extended the Snail protein half-life (Fig. 5D), and MG132 treatment increased Snail steady-state protein levels in HCT116 cells with or without EpEX treatment (Fig. 5E). EpEX also led to a decreased level of ubiquitylated Snail compared to that of the control cells (Fig. 5F).

In this regard, two consensus motifs in serine-rich regions of Snail (motif 1: S97, S101; motif 2: S108, S112, S116, S120) are crucial to its post-transcriptional regulation and ubiquitination-mediated proteasomal degradation [32]. HCT116 cells were transfected with wild-type (Snail-WT) and three mutant (Snail-2SA, -4SA, and -6SA) Snail constructs (Fig. 5G). While treatment of the transfected cells with EpEX significantly increased the expression of Snail-WT and -2SA, no such effects were seen on the expression of Snail-4SA and -6SA mutants (Fig. 5H). These data suggest that EpEX regulates the stability of Snail protein via the serine-rich consensus motif 2 of Snail in cancer cells.

### EpAb2-6 inhibits EpCAM and HGFR signaling and promotes active β-catenin and Snail protein degradation via activation of GSK3β

**Fig. 6:**
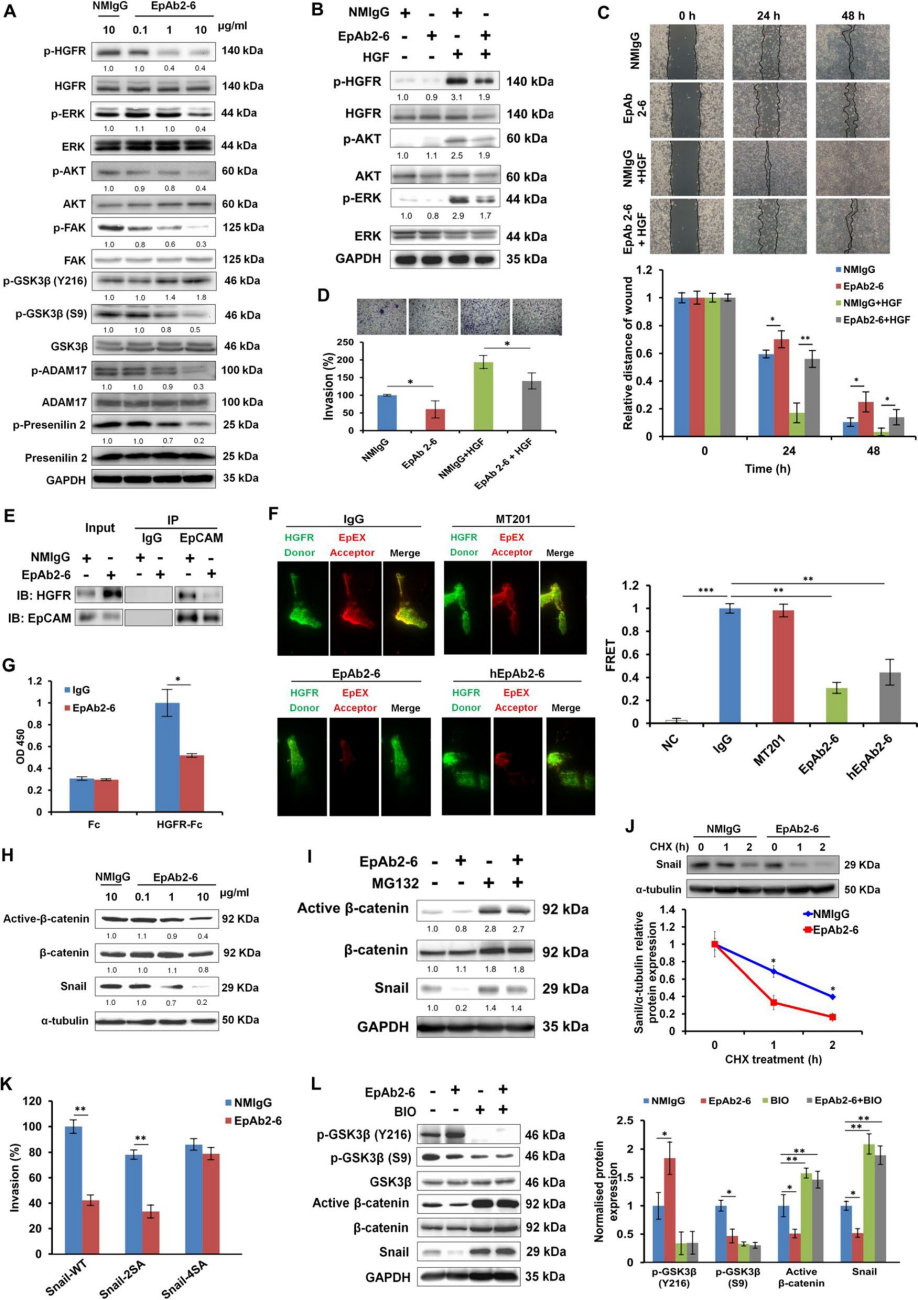
EpAb2-6 inhibits EpCAM and HGFR signaling and promotes active β-catenin and Snail protein degradation via activating GSK3β. **A** HCT116 cells were treated with 10 μg/ml control IgG (normal mouse IgG, NMIgG) or EpAb2-6 for 16 h, followed by treatment with EpEX-His (50 nM) for 15 min. Levels of phosphorylated HGFR, AKT, FAK, GSK3β, ERK, ADAM17, and presenilin 2 were examined by Western blotting. **B** HCT116 cells were treated with 10 μg/ml control IgG or EpAb2-6 for 16 h, followed by treatment without or with HGF (0.5 nM) for 15 min. Levels of phosphorylated HGFR, AKT, and ERK were examined by Western blotting. HCT116 cells were treated with EpAb2-6 (10 μg/ml) and HGF (0.5 nM). **C** Cells pre-treated with 5 µg/ml mitomycin-C for 4 h and then treated with IgG or EpAb2-6 and/or HGF. Cell migration was examined by the wound healing assay at the indicated times. **D** Cell invasion was assessed by a Transwell assay with matrigel after 24 h. **E** HCT116 cells were treated with NMIgG or EpAb2-6 (20 μg/ml) for 24 h and then immunoprecipitated with anti-EpCAM (IP: EpCAM) or anti-HGFR (IP: HGFR) antibodies, followed by Western blotting. **F** HCT116 cells were treated with NMIgG, MT201, EpAb2-6 or humanized EpAb2-6 (hEpAb2-6) (20 μg/ml) for 24 h. Representative images of TIRF-FRET experiments showing energy transfer from HGFR to EpEX in HCT116 cells. HGFR as donor channel (AF488) and EpEX as acceptor channel (AF568). Negative control (NC): Donor HGFR-AF488 and acceptor normal IgG-AF568. **G** EpEX-His (2 μg/ml) co-treated with 1 μg IgG or EpAb2-6 was added to HGFR-Fc-coated (1 μg/ml) ELISA plates and detected by TMB colorimetric peroxidase assay. **H** β-catenin and Snail protein levels were detected by Western blotting in HCT116 cells treated with NMIgG or EpAb2-6 for 24 h. **I** HCT116 cells were treated with 10 μM MG132 and EpAb2-6 for 6 h before cell collection and subsequent Western blotting. **J** Stability of Snail protein in HCT116 cells treated with NMIgG or EpAb2-6. Cells were treated with cyclohexamide (CHX) 100 μg/ml at the indicated intervals and subjected to Western blotting. Bottom graph shows quantification of Snail half-life in indicated groups. **K** Invasion assays were performed using HCT116 cells expressing Snail-WT, -2SA, or -4SA plasmids, with or without EpAb2-6 treatment. **L** Protein expression was analyzed by Western blotting in HCT116 cells after treatment with EpAb2-6 and 2 μM GSK3β inhibitor (BIO) for 24 h. Quantification of the normalized protein expression in the right panel. Statistical differences were determined by two-tailed Student *t* test. *N* = 3 independent experiments. All data are presented as mean ± SEM. *p < 0.05; **p < 0.01; **p < 0.001

Previously, we developed a neutralizing antibody called EpAb2-6, which targets EpEX and induces cancer cell apoptosis [5, 10]. Therefore, we used EpAb2-6 to block the function of EpEX in colon cancer cells and analyzed the phosphorylation levels of HGFR, AKT, FAK, GSK3β, ERK, ADAM17, and presenilin 2 in HCT116 cells. EpAb2-6 treatment results decreased phosphorylation of HGFR, AKT, ERK, and FAK compared to control IgG treatment (Fig. 6A). Additionally, HGF treatment increased phosphorylation levels of HGFR, AKT, and ERK in HCT116 cells, while the levels of these phosphorylated proteins were significantly decreased in cells treated with EpAb2-6 (Fig. 6B). Notably, the invasion and migration activities of HCT116 cells were also significantly reduced with EpAb2-6 treatment (Fig. 6C, D). When HCT116 cells were treated with HGF after EpAb2-6, the effects of EpAb2-6 on invasion and migration were partially blunted (Fig. 6C, D).

We also found that EpAb2-6 decreased the association between EpCAM and HGFR, as detected by IP of endogenous proteins in HCT116 cells (Fig. 6E). FRET imaging results further support the direct interaction between EpEX and HGFR; we could successfully observe the energy transfer from donor (HGFR) to acceptor (EpEX) in the control IgG and anti-EpCAM mAb MT201 pre-treatment groups while, EpAb2-6 and humanized EpAb2-6 pretreatment substantially decreased EpEX binding to HGFR, therefore the energy transfer efficiency were blocked (Fig. 6F). To evaluate whether recombinant EpEX directly binds to HGFR, we performed ELISA to probe the interaction between purified EpEX-His and HGFR-Fc protein. The results further confirmed the binding of EpEX to HGFR and indicated that EpAb2-6 could inhibit such binding (Fig. 6G).

Following these experiments, we analyzed the Snail and active β-catenin protein expression in HCT116 cells treated with control IgG or EpAb2-6. The results showed that EpAb2-6 treatment decreased the protein levels of Snail and active β-catenin (Fig. 6H). Furthermore, active β-catenin and Snail steady-state protein levels were reduced by treatment with EpAb2-6, while treatment with proteasome inhibitor (MG132) increased active β-catenin and Snail steady-state protein levels (Fig. 6I). In addition, EpAb2-6 shortened the Snail protein half-life, as shown in a cyclohexamide treatment assay (Fig. 6J). Moreover, we found that overexpression of mutant Snail-4SA restored invasion capacity upon EpAb2-6 treatment, but Snail-WT and Snail-2SA did not (Fig. 6K). These data show that EpAb2-6 inhibition of colon cancer invasion likely occurs as a result of suppressed EpCAM-HGFR axis signaling, which allows rapid Snail protein degradation.

Furthermore, EpAb2-6 decreased suppressive phosphorylation of GSK3β at S9 (inactive GSK3β), and it simultaneously increased activating phosphorylation of the protein at Y216 (active GSK3β). These changes are indicative of increased GSK3β activity and were coincident with the observed decreases in active β-catenin and Snail proteins. Correspondingly, active β-catenin and Snail proteins were increased after treatment with the GSK3β inhibitor, BIO, in EpAb2-6-treated or non-treated groups (Fig. 6L). Together, the results suggest that EpAb2-6 inhibits metastatic processes by downregulating HGFR signaling and allows active β-catenin and Snail protein degradation via increased GSK3β activity.

We further tested whether divalent antibody fragments F(ab′)_2_ of EpAb2-6 could bind to EpEX and induce apoptosis. Our experimental results showed that F(ab′)_2_ of EpAb2-6 could indeed bind to EpEX (Additional file 1: Fig. S6A) and induce apoptosis in colon cancer cells (Additional file 1: Fig. S6B). We also used an apoptosis assay to evaluate whether humanized EpAb2-6 (hEpAb2-6) and human anti-EpCAM antibody, adecatumumab (MT201), share similar activities. EpAb2-6 and hEpAb2-6 exhibited identical functionalities in inducing apoptosis, but MT201 did not show any such effects in HCT116 or HT29 cancer cells (Additional file 1: Fig. S6C). We also found that EpAb2-6 and hEpAb2-6 could both inhibit the phosphorylation of HGFR, AKT, and ERK, but MT201 did not (Additional file 1: Fig. S7A). Moreover, phosphorylated ADAM17 and presenilin 2 levels decreased after treatment with EpAb2-6 or hEpAb2-6, while treatment with the MT201 antibody had no such effects (Additional file 1: Fig. S7B).

### EpAb2-6 binds to the EGF-like domains I and II of EpCAM

A previous study identified the binding epitope of EpAb2-6 antibody as the LYD motif in EpCAM, which corresponds to amino acid residues 94–96; in particular, residue 95 (Y95) plays a major role in EpAb2-6 binding [10]. Here, we found that EpEX binds to HGFR through its EGF-like domains I and II (Fig. 1F, G), and EpAb2-6 can inhibit EpEX binding to HGFR (Fig. 5E, F). Therefore, we wanted to determine whether the antibody binds to EpCAM at both EGF-like domains of EpEX (Additional file 1: Fig. S8A–C). In order to confirm that EpAb2-6 recognizes the LYD motif in EpCAM, we constructed cDNA sequences encoding the first (aa 27–59; EGF-I domain) and second (aa 66–135; EGF-II/TY domain) EGF-like repeats of EpCAM. PCR-based site-directed mutagenesis was then used to introduce mutations into each domain (Additional file 1: Fig. S8D). The reactivity of EpAb2-6 antibody toward these EpCAM mutants was evaluated by immunofluorescence (Additional file 1: Fig. S8E), flow cytometry (Additional file 1: Fig. S8F), and cellular ELISA (Additional file 1: Fig. S8G). Amino acid mutations at EpCAM positions Y32 (EGF-I domain) or Y95 (EGF-II domain) caused marked reductions in EpAb2-6 binding but did not affect MT201 binding. Thus, we concluded that EpAb2-6 indeed binds to the EGF-I and EGF-II domains of EpEX, respectively, targeting amino acid residues Y32 and Y95.

### EpAb2-6 improves the efficacy of crizotinib therapy in a colon cancer animal model

**Fig. 7:**
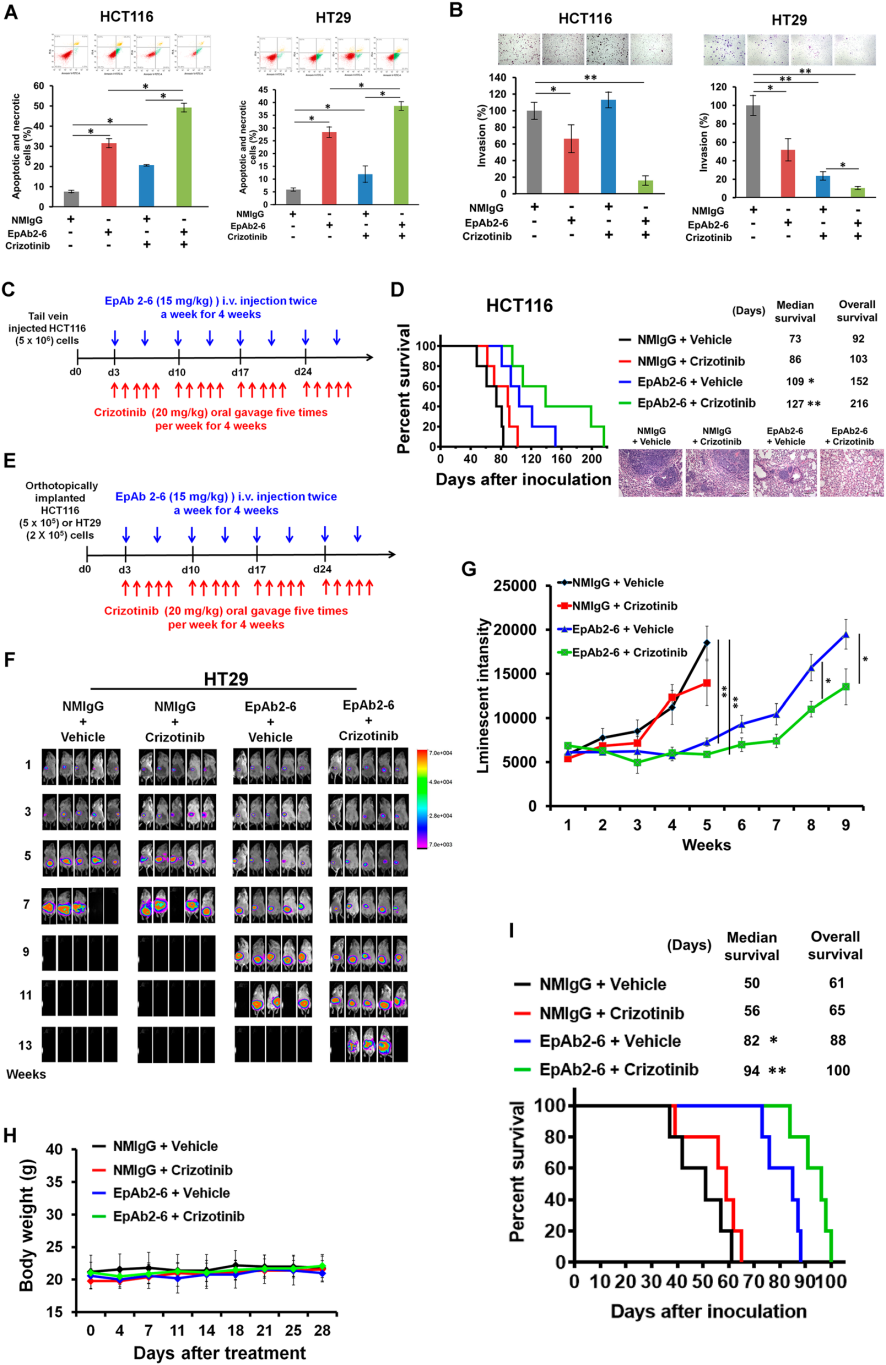

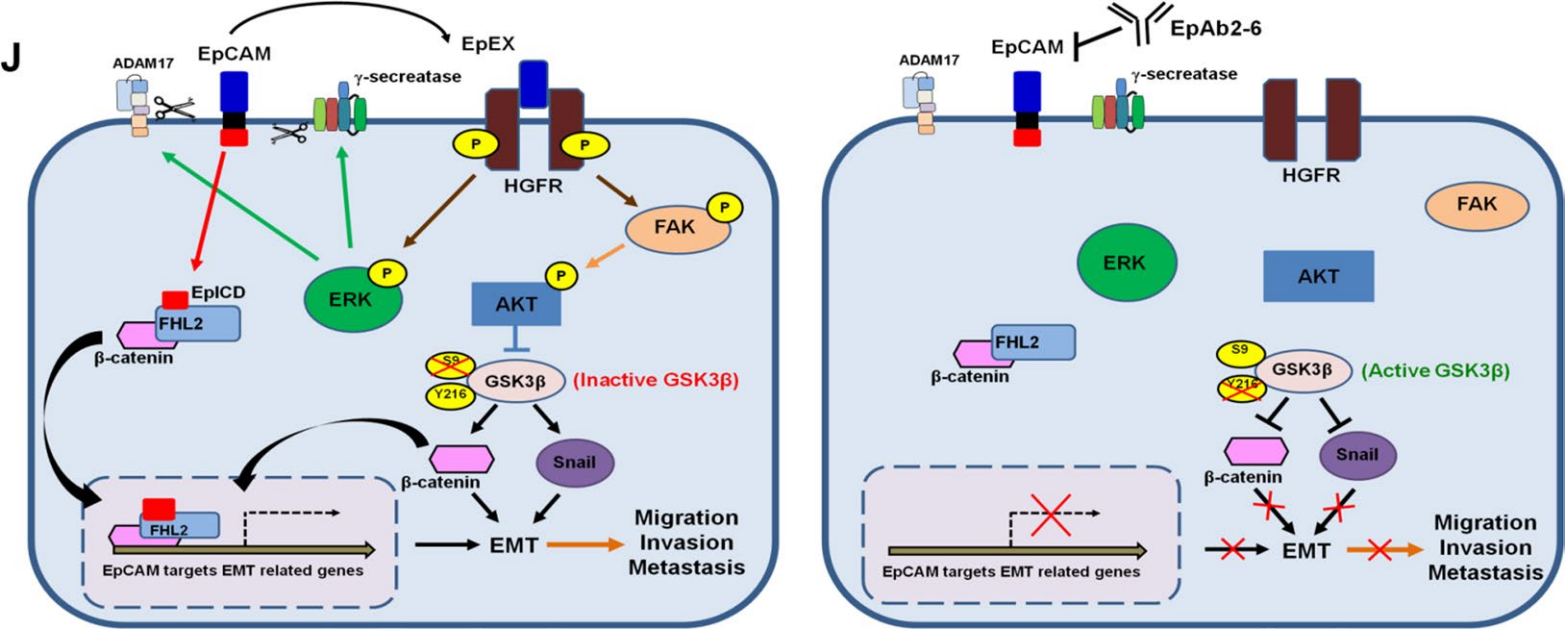
EpAb2-6 and crizotinib coordinately inhibit tumor progression and metastasis. **A** HCT116 and HT29 cells were treated with 10 μg/ml NMIgG or EpAb2-6 and 4 μM HGFR inhibitor crizotinib for 24 h. The apoptotic and necrotic cells were quantified by fluorescein annexin V-FITC/PI double labeling. **B** HCT116 and HT29 cells were treated with 10 μg/ml NMIgG or EpAb2-6 and 10 μM HGFR inhibitor crizotinib. Cell invasion was assessed by a Transwell assay with matrigel after 24 h. **C** Timeline of the experiment to evaluate EpAb2-6 and/or crizotinib effects in the metastatic animal model. **D** NOD/SCID mice were intravenously injected with 5× 106 HCT116 cells, followed by treatment with either control IgG, EpAb2-6 and/ or crizotinib. The survival curve, median survival days and representative H&E staining of lung tissues in metastatic animal models are shown. **E** Timeline of the experiment to evaluate EpAb2-6 and/or crizotinib in the orthotopic animal model. **F** NOD/SCID mice received orthotopic implantation of HT29-Luc cells and then were treated with control IgG (normal mouse IgG, NMIgG), crizotinib, EpAb2-6, or crizotinib combined with EpAb2-6 starting at 3 days after tumor inoculation. Tumor growth was monitored by examining bioluminescence with the IVIS 200 Imaging System. **G** HT29-Luc tumor cells monitored by bioluminescence quantification. **H** Body-weights of each treatment group in the HT29 orthotopic animal model after indicated treatments. **I** Survival curves and median survival days of each treatment group in the HT29 orthotopic animal model. **J** Summary illustration of the cell signaling events mediating EpCAM tumorigenic effects. In brief, EpEX binds to HGFR then stimulates HGFR to induce ERK and FAK-AKT activation, which promotes active β-catenin and Snail protein stabilization via reducing GSK3β activity that drives tumor progression, migration, and invasion. The EpCAM neutralizing antibody EpAb2-6 inhibits cancer cell invasion by blocking EpEX-HGFR axis mediated downstream signaling to promote reduction of active β-catenin and Snail protein stability. Statistical differences were determined by two-tailed Student *t* test. *N* = 5 independent experiments. All data are presented as mean ± SEM. *p < 0.01

Our data suggested that EpEX and HGFR coordinately stimulate downstream HGFR signaling to promote tumor progression and cell invasion, so we wanted to further test the anti-tumor effects of simultaneously blocking both EpCAM and HGFR signaling. Notably, we found that HGFR inhibitor crizotinib could enhance the apoptotic effects of EpAb2-6 on HCT116 and HT29 cancer cells (Fig. 7A). In the cell invasion assay, crizotinib also enhanced the inhibitory effects of EpAb2-6 on invasion in HCT116 and HT29 cells, as compared to control IgG (Fig. 7B).

To determine whether EpAb2-6 improves the efficacy of crizotinib therapy in colon cancer animal model, we first examined the effect of crizotinib and EpAb2-6 in the colon cancer cell HCT116 metastasis model. As illustrated in Fig. 7C, NOD/SCID mice were injected intravenously with HCT116 cells and then co-treated with crizotinib and EpAb2-6, or an equivalent volume of control IgG, at 3 days after cell injection. As a result, the median and overall survival times of HCT116-implanted mice that received the combination of EpAb2-6 and crizotinib were increased compared to the control IgG group (Fig. 7D), supporting the idea that EpAb2-6 can improve the anti-metastatic action of crizotinib in vivo.

Next, we tested the combined effects of EpAb2-6 and crizotinib as a therapeutic strategy in an orthotopic mouse model of colon cancer. As illustrated in Fig. 7E, tumor growth was assessed by in vivo monitoring of HCT116-Luc and HT29-Luc cells, which stably express firefly luciferase. Before initiation of the therapeutic treatment (3 days after tumor cell implantation), tumor growth could be observed in all mice. After treatment, bioluminescence intensities in mice receiving EpAb2-6 or the combination of EpAb2-6 and crizotinib were significantly decreased compared to the control IgG or crizotinib alone groups; similar effects were observed in orthotopic models transplanting HCT116 (Additional file 1: Fig. S9A, B) or HT29 (Fig. 7F, G) cells. Moreover, the body weights were not significantly different between treatment groups in either the HCT116 (Additional file 1: Fig. S9C) or HT29 (Fig. 7H) orthotopic transplantation models. The median and overall survival times of mice transplanted with HCT116 (Additional file 1: Fig. S9D) or HT29 (Fig. 7I) cells receiving the combination of EpAb2-6 and crizotinib were significantly increased compared to the control IgG groups. In summary, the results of our experiments on metastatic and orthotopic animal models showed that all animals in the control IgG and crizotinib groups developed significant tumors and had poor survival. Meanwhile, the EpAb2-6-treated group had much slower tumor progression and showed higher median survival than the control IgG- or crizotinib-treated groups. Notably, the attenuation of tumor progression was most pronounced in the combination treatment group.

## Discussion

EpCAM expression is correlated with tumorigenesis and metastasis in many cancers, so we sought to elucidate the underlying mechanisms in this study. Here, we show that EpEX-induced tumor progression and metastasis are mediated by HGFR signaling. This major finding aligns with previous studies that have associated high HGFR expression or activation and poor outcome in cancer patients [33]. For example, high expression of HGFR is indicative of poor prognosis in thyroid carcinoma and non-small cell lung cancer (NSCLC). In addition, it is a predictor of tumor invasion and lymph node metastases in colon cancer [34, 35]. Previous reports have also shown that in models of gastric cancer and CRC, blockade of HGFR signaling can reduce tumor cell growth and spread in vitro and in vivo [36–38].

HGF is a cytokine that can modulate the proliferation of epithelial cells, and it is mainly expressed and secreted by mesenchymal cells [39, 40]. The major coordinator of HGF signaling is HGFR, and the complex program induced by this signaling pathway promotes proliferation, survival, matrix degradation, and migration. Together HGFR and HGF form the basis for an essential epithelial and mesenchymal interaction necessary for wound closure and angiogenesis [41]. Our results show that EpCAM knockout attenuates phosphorylation of HGFR in colon cancer cells, and the cell growth and migration capacities in EpCAM knockout HCT116 cells were significantly reduced compared to wild-type cells. The ability of EpCAM to regulate HGFR signaling suggests that this pathway may play an important role in regulating the progression of colon cancer.

Tyrosine kinase inhibitors (TKIs) are small molecule drugs that can target activated RTKs regardless of ligand presence by preventing ATP from reaching the ATP-binding pocket of the kinase domain [42]. Typically, drug resistance arises due to the acquisition of mutations in the RTK that can abolish the effect of the TKI or by amplifying another RTK that can stimulate similar signaling, such as HGFR [43]. Previously our group used a Human Phospho-RTK Array Kit to screen for phosphorylation of RTKs in EpEX-treated HCT116 colon cancer cells. This previous study showed that HGFR and EGFR-tyrosine phosphorylation was stimulated by EpEX treatment [5]. In the current study, we found that HCT116 colon cancer cells treated with EpEX induce HGFR phosphorylation. Interestingly, HGFR tyrosine kinase inhibitor (SU11274) could attenuate EpEX-mediated ERK and AKT phosphorylation. Furthermore, we confirmed that depletion of HGFR could attenuate EpEX-induced HGFR and its downstream signaling, cell growth, migration, and invasion, consistent with the effects of the HGFR inhibitor.

Many studies have shown that EMT is associated with cancer progression and metastasis [44]. EMT affords tumors with stem cell-like plastic characteristics required for acquiring mesenchymal features, allowing tumor cells to disseminate and become more invasive [45, 46]. Indicators of EMT include increased expression of mesenchymal markers, such as Vimentin, Snail, and Slug, alongside decreased expression of epithelial markers like E-cadherin, which disrupts cell–cell junctions [47]. Many reports have shown that EMT in different cancer types can promote resistance to various therapeutic drugs [48–50]. Blocking EMT for therapeutic purposes may be accomplished by targeting the components of the tumor microenvironment that contribute to the activation of the EMT program in tumor cells [22]. For example, HGF induces the EMT program via HGFR signaling, thereby enhancing the invasive and metastatic potential of cancer cells by allowing the cells to survive in the bloodstream without anchorage. Previous reports indicated FAK-PI3K/AKT and MAPK signaling pathways promoting migration and metastasis in colon cancer and glioblastoma [51]. Our data showed that inhibitors (i.e., SU11274, LY294002, U0126, or PF-562271) of these molecules can attenuate EpEX-induced migration and invasion in HCT116 cells to different degrees. Therefore, EpCAM signaling appears to be involved in activation of these tyrosine kinases.

Inhibition of GSK3β by EpEX signaling can stabilize both β-catenin and Snail, which coordinately induce EMT-associated cell migration and invasion. Of note, EMT is correlated with high expression of non-phosphorylated (active) β-catenin and translocation of β-catenin into the nucleus. Still, the overexpression of β-catenin alone does not necessarily promote EMT-associated processes [32]. Additionally, Snail is a zinc-finger transcription factor that triggers EMT by repressing E-cadherin expression. Many oncogenic signals, such as PI3K/AKT, MAPK, and Wnt, have been shown to inhibit GSK3β and thus cause the stabilization of Snail and subsequent EMT [32]. Our data showed that EpEX induces EMT and invasion by stabilizing active β-catenin and Snail via decreased GSK3β activity. Furthermore, our anti-EpCAM antibody inhibits EMT and invasion by increasing GSK3β activity, which leads to the degradation of active β-catenin and Snail.

HGFR signaling is an important target for anticancer therapy, and substantial efforts have been made to develop antagonists of this pathway. Many small-molecule inhibitors of the HGFR tyrosine-kinase domain are currently being evaluated in clinical trials. HGFR overexpression is known to promote drug resistance in many cancer cells, resulting in poor treatment efficacy and shortened patient survival time [52]. For example, strong preclinical and clinical evidence shows that the HGFR signaling pathway is a crucial driver of multidrug resistance in multiple myeloma patients [53]. Currently, two non-selective HGFR TKIs have been approved: crizotinib (first approved in 2011) for ALK- and ROS1-positive NSCLC and cabozantinib (First approved in 2016) for thyroid cancer and kidney cancer [54]. The relevance of HGFR inhibition is under intense evaluation, with several ongoing clinical trials on crizotinib. Among the potential treatments for NSCLC, crizotinib and other HGFR-targeting therapies have some of the most beneficial outcomes. This fact underscores the importance of deeply understanding the mechanisms that can be used to block HGFR activation. Furthermore, we found that EpCAM/EpEX can induce the HGFR-ERK-AKT signaling axis. According to these findings, EpCAM might be an excellent target for combination therapies with crizotinib. Indeed, in our experiments, EpAb2-6 decreased the level of phosphorylated HGFR and improved the therapeutic efficacy of crizotinib in animal models. Thus, our findings reveal a novel action of EpCAM in regulating HGFR signaling and suggest a new strategy for EpCAM/HGFR-targeted combination therapy.

Our group has produced an EpEX-neutralizing antibody, EpAb2-6, which can block the function of EpEX. EpAb2-6 treatment is known to disrupt signaling in the EpEX/EGFR/ADAM17 axis, which comprises a positive feedback loop to promote EpCAM cleavage and subsequently increase EpEX and EpICD production [5, 10]. In this study, we found decreased phosphorylated HGFR was observed after EpAb2-6 treatment. Furthermore, EpAb2-6 could attenuate the invasion and migration capacity of colorectal cancer cells. We confirmed that after crizotinib treatment, colorectal cancer cells were sensitized to EpAb2-6-induced apoptosis (Fig. 7A). Our results from the metastatic and orthotopic colon cancer animal model also indicated that tumor growth was significantly inhibited after combined treatment of EpAb2-6 and crizotinib (Fig. 7C–I; Additional file 1: Fig. S9).

To the best of our knowledge, this is the first study to demonstrate that EpCAM/EpEX binds to HGFR and induces tumor progression and metastasis through ERK and FAK-AKT by inducing HGFR activation and GSK3β-Snail and β-catenin signaling in colon cancer cells. We further demonstrate that inhibition or depletion of EpCAM signaling leads to decreases in HGFR activation and its downstream signaling (Fig. 7J). Treatment with anti-EpCAM mAb EpAb2-6 reduced colon cancer progression and metastasis, and importantly, it improved the survival of mice in orthotopic tumor and metastasis models.

## Conclusion

EpEX binds to HGFR and induces tumor progression and metastasis through ERK and FAK-AKT by inducing HGFR activation and GSK3β-Snail and β-catenin signaling in colon cancer cells. The therapeutic antibodies targeting EpCAM in combination with HGFR inhibitors may hold great potential for cancer patients with high EpCAM expression. The insights gained from these findings may be helpful in the development of novel anticancer therapeutics that can inhibit metastasis and improve patient outcomes.

### Supplementary Information

**Additional file 1: Figure S1.** Cell invasion and phosphorylation of HGFR, AKT, and ERK are partially reversed in EpCAM knockout HT29 cells after EpEX treatment. **Figure S2.** EpCAM deficiency suppresses EMT in colon cancer cells. **Figure S3.** EpEX could induce EMT and invasion without EGFR. **Figure S4.** EpEX can promotes invasion via HGFR signaling pathway. **Figure S5.** EpCAM regulates EMT-related genes expression. **Figure S6.** EpAb2-6 binds to EpEX and induces apoptosis via F(ab′)2. **Figure S7.** EpAb2-6 inhibits regulated intramembrane proteolysis (RIP) activation and HGFR signaling. **Figure S8.** EpAb2-6 binds to both EGF-like domain I and II of EpCAM. **Figure S9.** EpAb2-6 and crizotinib coordinately inhibit tumor progression in the HCT116 orthotopic colon cancer animal model. **Table S1.** List of oligonucleotides for real-time PCR assay.

## Data Availability

Not applicable.

## Abbreviations

ADAM17: A disintegrin and metalloprotease 17
CRC: Colorectal cancer
EGF: Endothelial growth factor
EGFR: Endothelial growth factor receptor
ELISA: Enzyme-linked immunosorbent assay
EMT: Epithelial–mesenchymal transition
EpCAM: Epithelial cell adhesion molecule
EpEX: Extracellular domain of EpCAM
EpICD: Intra-cellular domain of EpCAM
ERK: Extracellular signal-regulated kinase
FAK: Focal adhesion kinase
FRET: Fluorescence resonance energy transfer
HGF: Hepatocyte growth factor
HGF: Hepatocyte growth factor
HGFR: Hepatocyte growth factor receptor
IP: Immunoprecipitation
KO: Knockout
MAPK: Mitogen-activated protein kinase
qRT-PCR: Quantitative real-time polymerase chain reaction
RIP: Regulated intramembrane proteolysis
RTK: Receptor tyrosine kinase
TIRF: Total internal reflection fluorescence
TKIs: Tyrosine kinase inhibitors
