# Correction: Epithelial cell adhesion molecule (EpCAM) regulates HGFR signaling to promote colon cancer progression and metastasis

**DOI:** 10.1186/s12967-023-04797-x

**Published:** 2024-01-08

**Authors:** Chi-Chiu Lee, Chia-Jui Yu, Sushree Shankar Panda, Kai-Chi Chen, Kang-Hao Liang, Wan-Chen Huang, Yu-Shiuan Wang, Pei-Chin Ho, Han-Chung Wu

**Affiliations:** 1 Institute of Cellular and Organismic Biology, Academia Sinica, 128 Academia Road, Section 2, Nankang, Taipei 11529 Taiwan; 2 Biomedical Translation Research Center (BioTReC), Academia Sinica, Taipei, 11529 Taiwan

**Correction** **:** **Journal of Translational Medicine (2023) 21:530** 10.1186/s12967-023-04390-2

Following publication of the original article [1], we have been notified about the errors in Fig. 7A, B, G. In Fig. 7A, B, the label of NMIgG should be revise to “–” in the EpAb2-6 and Crizotinib combine treatment group. In Fig. 7G, the red curve is NMIgG + Crzotinib and the blue curve is EpAb2-6 + Vehicle. 

The incorrect version of Fig. 7 is as per below:
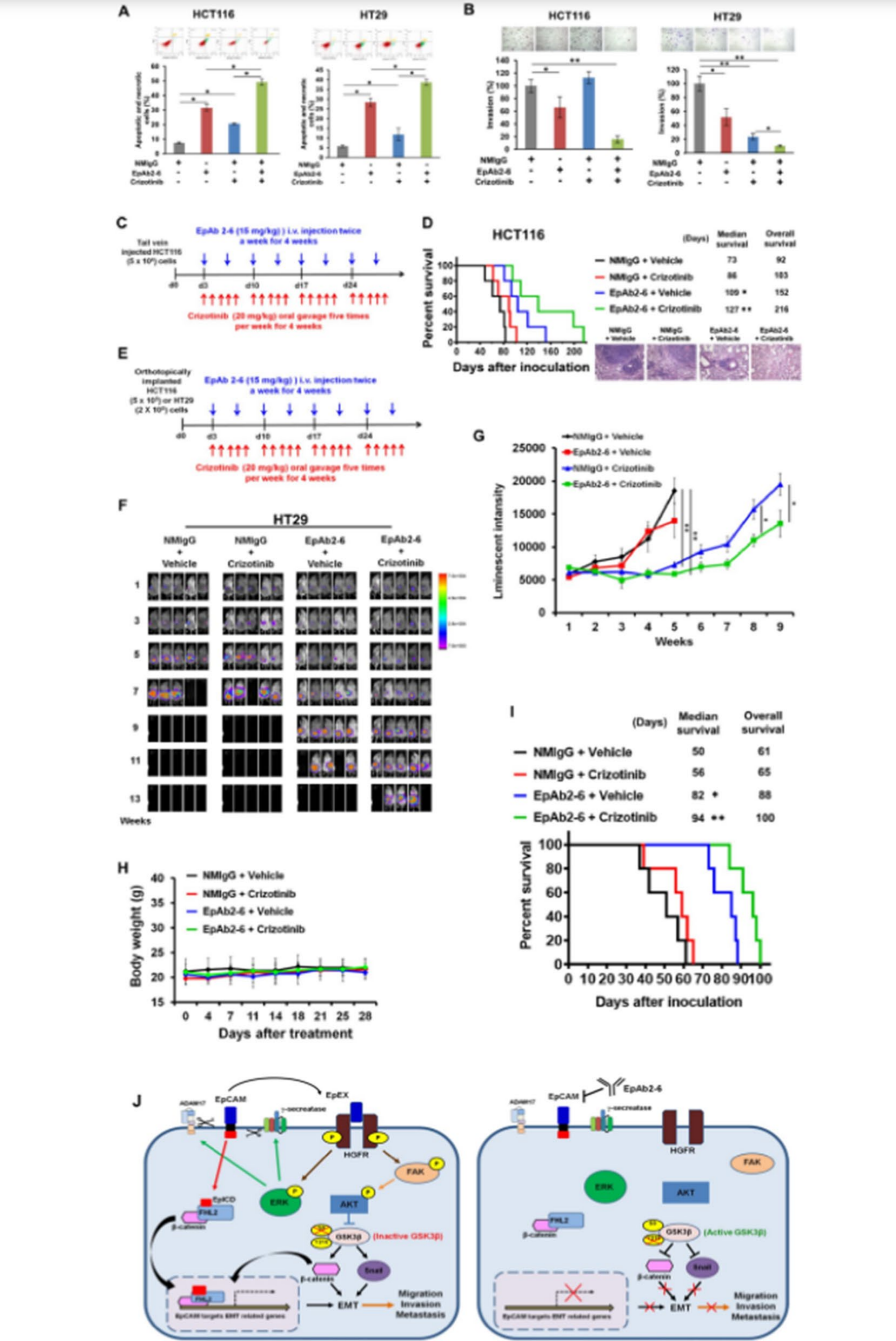


**Fig. 7** EpAb2-6 and crizotinib coordinately inhibit tumor progression and metastasis. **A** HCT116 and HT29 cells were treated with 10 μg/ml NMIgG or EpAb2-6 and 4 μM HGFR inhibitor crizotinib for 24 h. The apoptotic and necrotic cells were quantified by fluorescein annexin V-FITC/PI double labeling. **B** HCT116 and HT29 cells were treated with 10 μg/ml NMIgG or EpAb2-6 and 10 μM HGFR inhibitor crizotinib. Cell invasion was assessed by a Transwell assay with matrigel after 24 h. **C** Timeline of the experiment to evaluate EpAb2-6 and/or crizotinib effects in the metastatic animal model. **D** NOD/SCID mice were intravenously injected with 5 × 10^6^ HCT116 cells, followed by treatment with either control IgG, EpAb2-6 and/ or crizotinib. The survival curve, median survival days and representative H&E staining of lung tissues in metastatic animal models are shown. **E** Timeline of the experiment to evaluate EpAb2-6 and/or crizotinib in the orthotopic animal model. **F** NOD/SCID mice received orthotopic implantation of HT29-Luc cells and then were treated with control IgG (normal mouse IgG, NMIgG), crizotinib, EpAb2-6, or crizotinib combined with EpAb2-6 starting at 3 days after tumor inoculation. Tumor growth was monitored by examining bioluminescence with the IVIS 200 Imaging System. **G** HT29-Luc tumor cells monitored by bioluminescence quantification. **H** Body-weights of each treatment group in the HT29 orthotopic animal model after indicated treatments. **I** Survival curves and median survival days of each treatment group in the HT29 orthotopic animal model. J Summary illustration of the cell signaling events mediating EpCAM tumorigenic effects. In brief, EpEX binds to HGFR then stimulates HGFR to induce ERK and FAK-AKT activation, which promotes active β-catenin and Snail protein stabilization via reducing GSK3β activity that drives tumor progression, migration, and invasion. The EpCAM neutralizing antibody EpAb2-6 inhibits cancer cell invasion by blocking EpEX-HGFR axis mediated downstream signaling to promote reduction of active β-catenin and Snail protein stability. Statistical differences were determined by two-tailed Student t test. N = 5 independent experiments. All data are presented as mean ± SEM. *p < 0.01

The correct version of Fig. 7 is as per below:
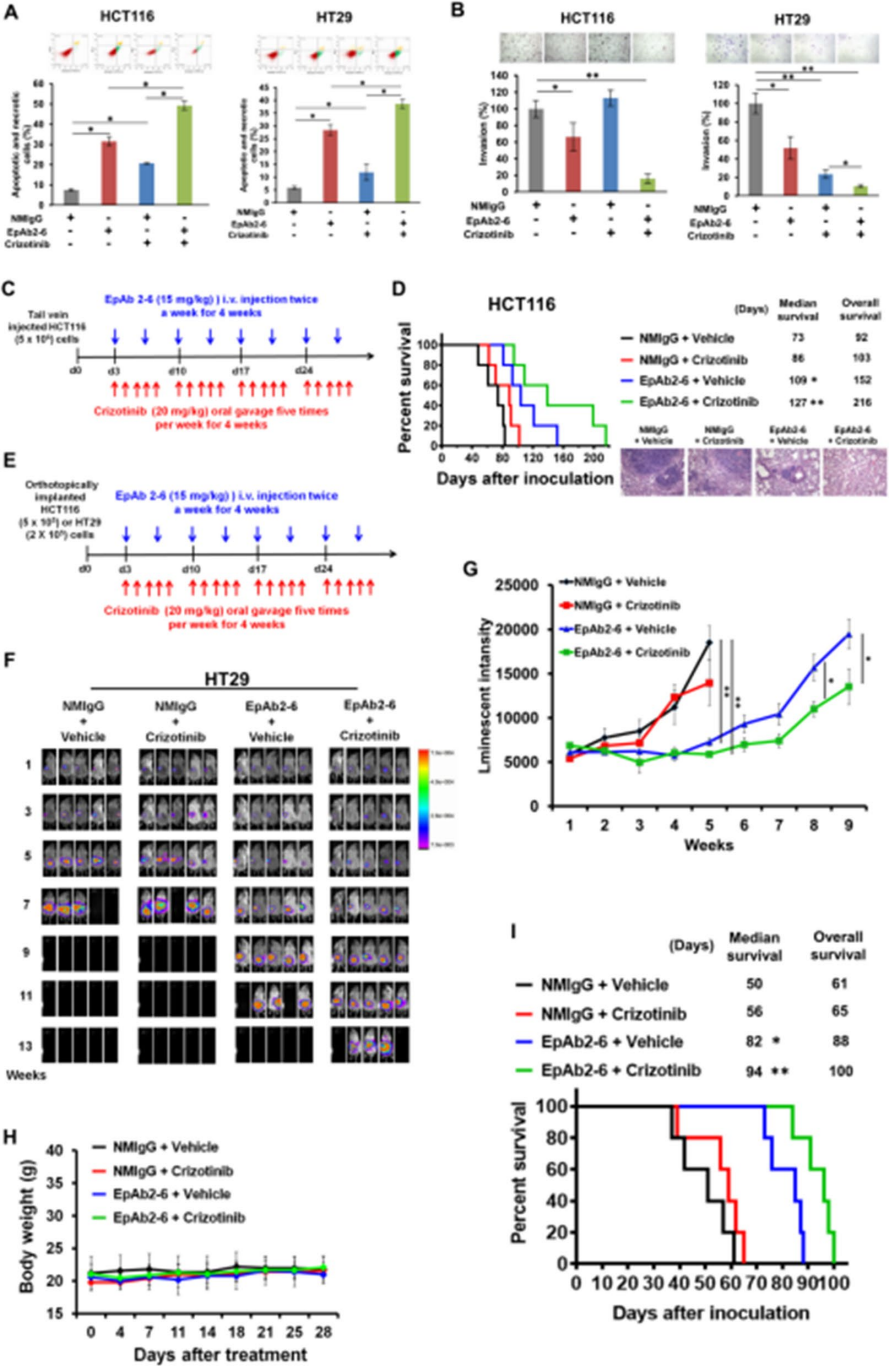


**Fig. 7**. EpAb2-6 and crizotinib coordinately inhibit tumor progression and metastasis. **A** HCT116 and HT29 cells were treated with 10 μg/ml NMIgG or EpAb2-6 and 4 μM HGFR inhibitor crizotinib for 24 h. The apoptotic and necrotic cells were quantified by fluorescein annexin V-FITC/PI double labeling. **B** HCT116 and HT29 cells were treated with 10 μg/ml NMIgG or EpAb2-6 and 10 μM HGFR inhibitor crizotinib. Cell invasion was assessed by a Transwell assay with matrigel after 24 h. **C** Timeline of the experiment to evaluate EpAb2-6 and/or crizotinib effects in the metastatic animal model. **D** NOD/SCID mice were intravenously injected with 5 × 10^6^HCT116 cells, followed by treatment with either control IgG, EpAb2-6 and/ or crizotinib. The survival curve, median survival days and representative H&E staining of lung tissues in metastatic animal models are shown. **E** Timeline of the experiment to evaluate EpAb2-6 and/or crizotinib in the orthotopic animal model. **F** NOD/SCID mice received orthotopic implantation of HT29-Luc cells and then were treated with control IgG (normal mouse IgG, NMIgG), crizotinib, EpAb2-6, or crizotinib combined with EpAb2-6 starting at 3 days after tumor inoculation. Tumor growth was monitored by examining bioluminescence with the IVIS 200 Imaging System. **G** HT29-Luc tumor cells monitored by bioluminescence quantification. **H** Body-weights of each treatment group in the HT29 orthotopic animal model after indicated treatments. **I** Survival curves and median survival days of each treatment group in the HT29 orthotopic animal model. **J** Summary illustration of the cell signaling events mediating EpCAM tumorigenic effects. In brief, EpEX binds to HGFR then stimulates HGFR to induce ERK and FAK-AKT activation, which promotes active β-catenin and Snail protein stabilization via reducing GSK3β activity that drives tumor progression, migration, and invasion. The EpCAM neutralizing antibody EpAb2-6 inhibits cancer cell invasion by blocking EpEX-HGFR axis mediated downstream signaling to promote reduction of active β-catenin and Snail protein stability. Statistical differences were determined by two-tailed Student t test. N = 5 independent experiments. All data are presented as mean ± SEM. *p < 0.01

We were also notified that there was incorrectly description in the text body of the article (‘Material and methods’ section).

It is now:

To observe the Wnt HGFR-EpEX interaction, the cells were fixed and co-stained with HGFR or EpEX antibodies as described earlier in the IFS with cell lines section.

It should be:

To observe the HGFR-EpEX interaction, the cells were fixed and co-stained with HGFR or EpEX antibodies as described earlier in the IFS with cell lines section.

The original article was updated.
