# Supplementary material for: Epithelial cell adhesion molecule (EpCAM) regulates HGFR signaling to promote colon cancer progression and metastasis
Source: J Transl Med. 2023 Aug 5;21:530. doi: 10.1186/s12967-023-04390-2 (PMC10404369; doi:10.1186/s12967-023-04390-2)
Supplement: Supplementary file 1 — Additional file 1: Figure S1. Cell invasion and phosphorylation of HGFR, AKT, and ERK are partially reversed in EpCAM knockout HT29 cells after EpEX treatment. Figure S2. EpCAM deficiency suppresses EMT in colon cancer cells. Figure S3. EpEX could induce EMT and invasion without EGFR. Figure S4. EpEX can promotes invasion via HGFR signaling pathway. Figure S5. EpCAM regulates EMT-related genes expression. Figure S6. EpAb2-6 binds to EpEX and induces apoptosis via F(ab′)2. Figure S7. EpAb2-6 inhibits regulated intramembrane proteolysis (RIP) activation and HGFR signaling. Figure S8. EpAb2-6 binds to both EGF-like domain I and II of EpCAM. Figure S9. EpAb2-6 and crizotinib coordinately inhibit tumor progression in the HCT116 orthotopic colon cancer animal model. Table S1. List of oligonucleotides for real-time PCR assay. [file 12967_2023_4390_MOESM1_ESM.pdf]

Additional file 1

Epithelial cell adhesion molecule (EpCAM) regulates HGFR signaling to promote colon cancer progression and metastasis

By Lee et al.

Corresponding Author: Han-Chung Wu, Email: hcw0928@gate.sinica.edu.tw

Table of Contents:

Figures S1–S9 with their legends

Table S1

Fig. S2. Lee et al.

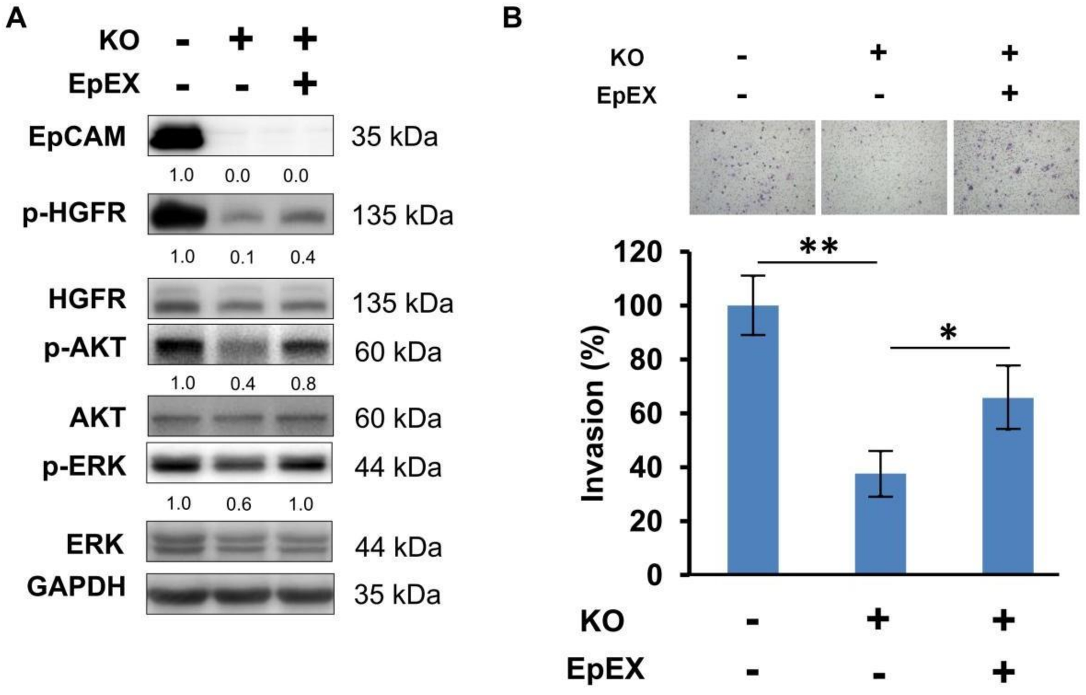

Fig. S1. Cell invasion and phosphorylation of HGFR, AKT, and ERK are partially reversed in EpCAM knockout HT29 cells after EpEX treatment. Wild-type (WT) or EpCAM knockout (KO) HT29 cells after EpEX treatment. (A) The levels of phosphorylated HGFR, AKT and ERK were assayed by Western blotting. (B) Cell invasion was examined by a Transwell chamber assay with matrigel. Statistical differences were determined by two-tailed Student *t* test. $N = 3$ independent experiments. All data are presented as mean ± SEM. *, $p < 0.05$; **, $p < 0.01$.

Fig. S3. Lee et al.

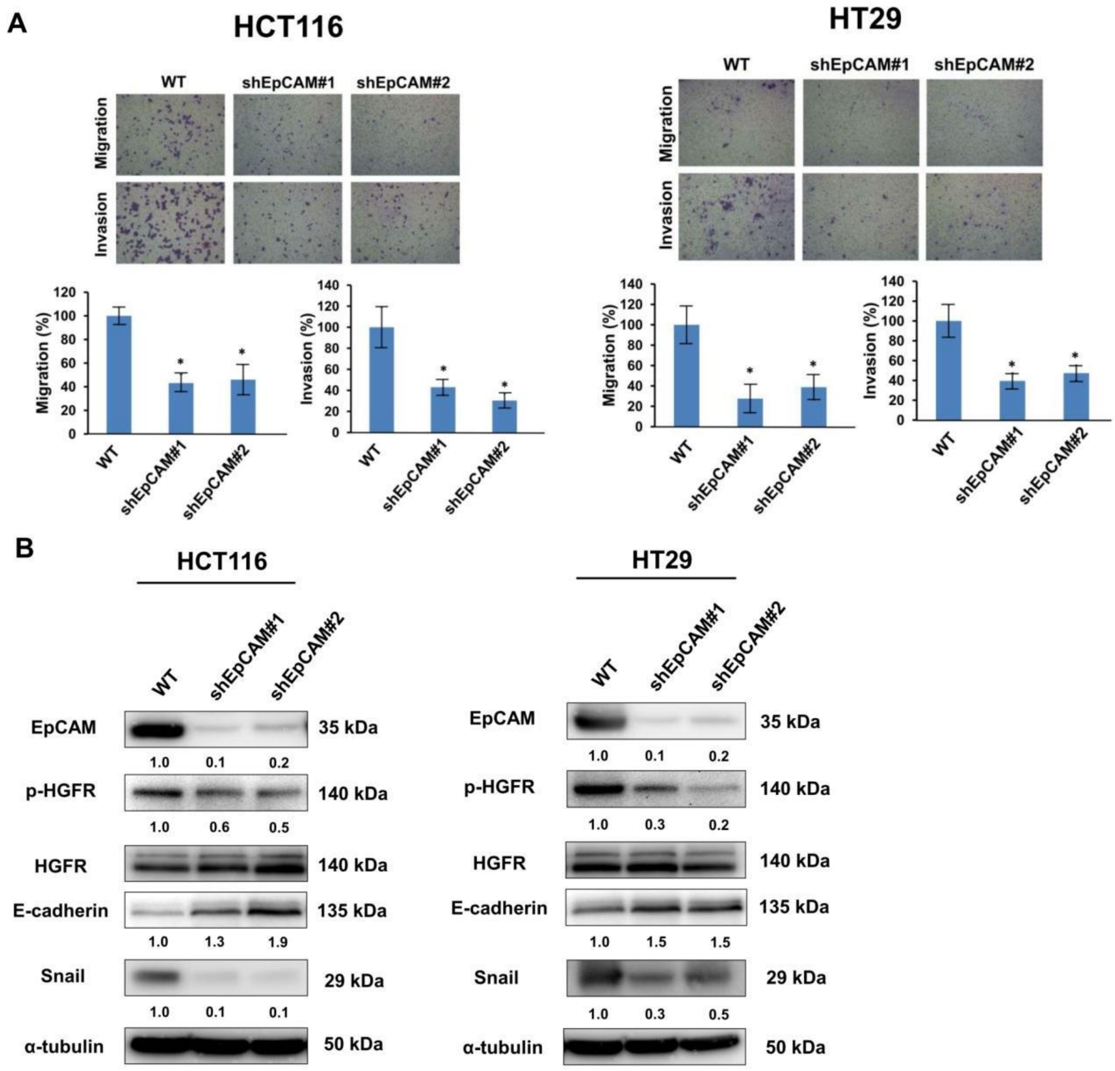

Fig. S2. EpCAM deficiency suppresses EMT in colon cancer cells.

(A) The effect of knocking down EpCAM on migration and invasion in HCT116 and HT29 cells was examined by Transwell chamber assay without or with matrigel. (B) The expression of EMT-related proteins (E-cadherin and Snail) in EpCAM knockdown HCT116 and HT29 cells. Statistical differences were determined by two-tailed Student *t* test. *N* = 3 independent experiments. All data are presented as mean ± SEM. *, $p < 0.05$.

Fig. S4. Lee et al.

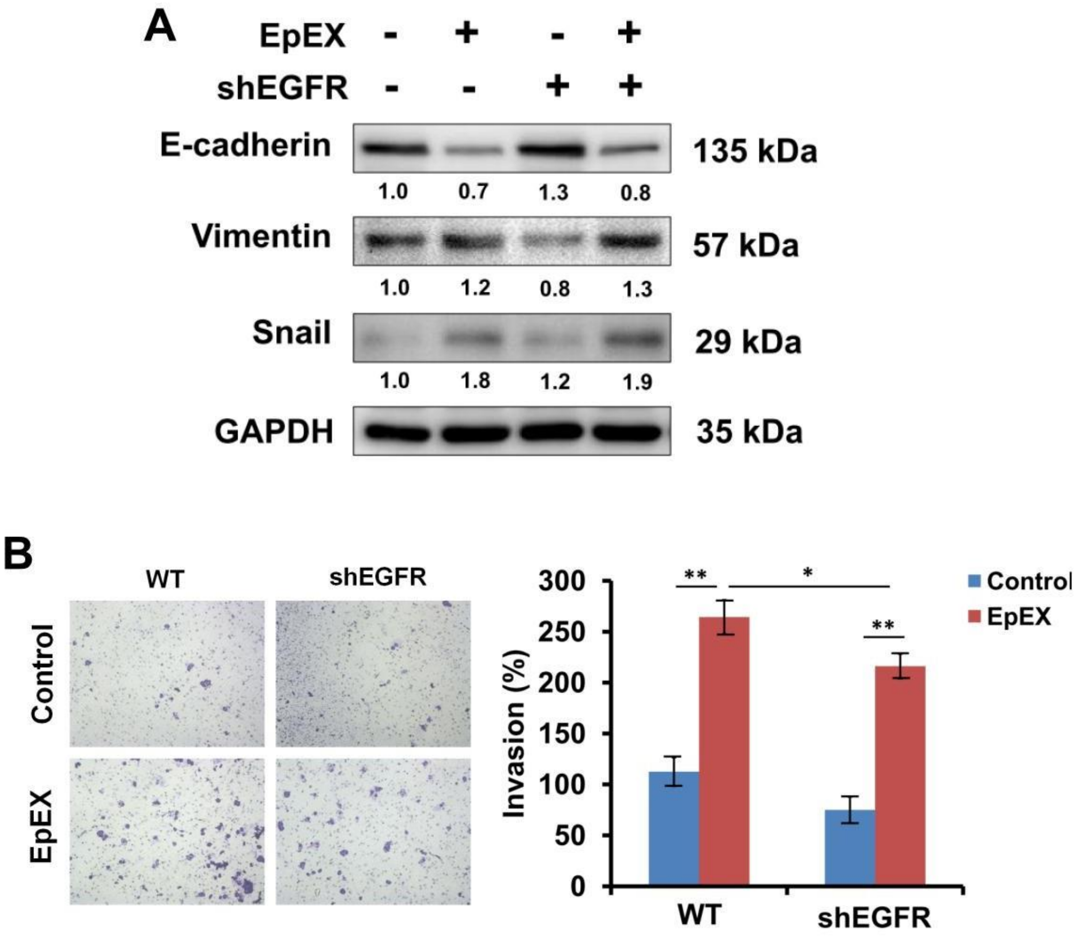

Fig. S3. EpEX could induce EMT and invasion without EGFR. (A)HCT116 cells after shLuc and shEGFR treatment were treated with 50 nM of EpEX-His. HCT116 cells after shLuc and shEGFR treatment were treated with 50 nM of EpEX-His with 2% FBS for 24h. EMT-related protein expression (E-cadherin, Vimentin, and Snail) was examined by Western blotting. (B) Cell invasion was examined by Transwell chamber assay with matrigel. Statistical differences were determined by two-tailed Student *t* test. *N* = 3 independent experiments. All data are presented as mean ± SEM. *, $p < 0.05$; **, $p < 0.01$.

Fig. S5. Lee et al.

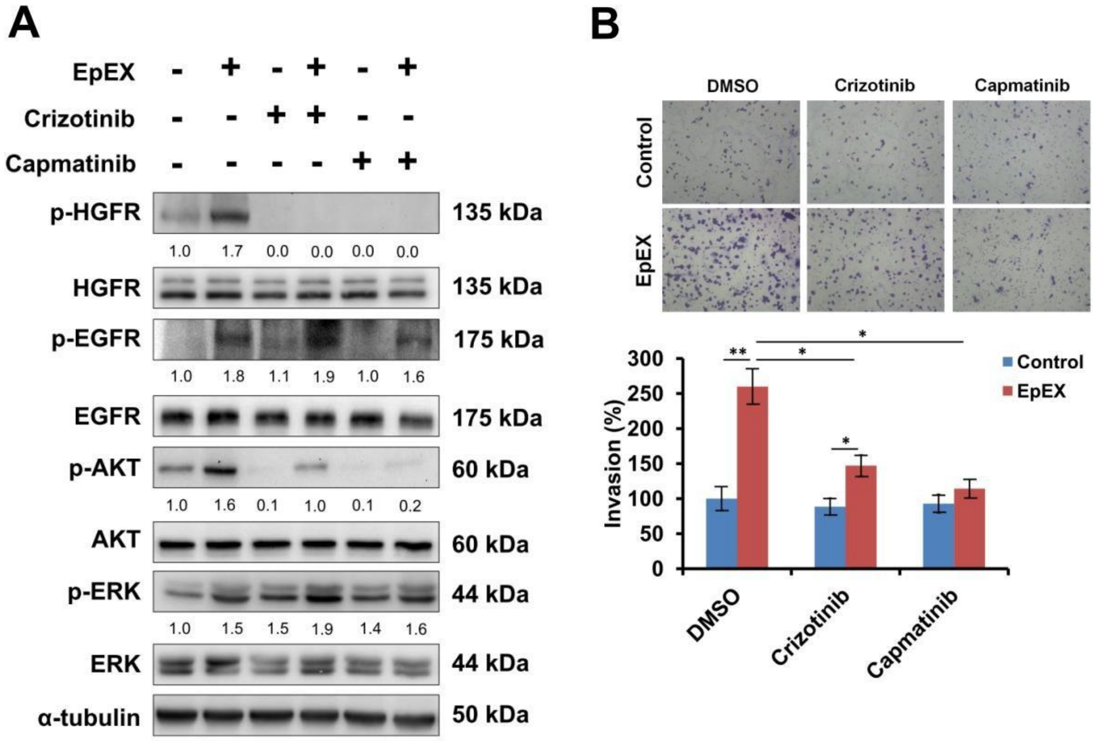

Fig. S4. EpEX can promotes invasion via HGFR signaling pathway. (A) Starved HCT116 were treated with HGFR inhibitor crizotinib (2 μM) and capmatinib (10 μM) for 1 h then treated with 50 nM of EpEX-His for 15 min. Thelevels of phosphorylated HGFR, EGFR, AKT, and ERK were examined by Western blotting. (B) HCT116 cells were treated with HGFR inhibitor crizotinib (2 μM) or capmatinib (10 μM) for 1 h then treated with 50 nM of EpEX-His for 24 h. Cell invasion was examined by Transwell chamber assay with matrigel. Statistical differences were determined by two-tailed Student *t* test. $N = 3$ independent experiments. All data are presented as mean ± SEM. *, $p < 0.05$; **, $p < 0.01$.

Fig. S6. Lee et al.

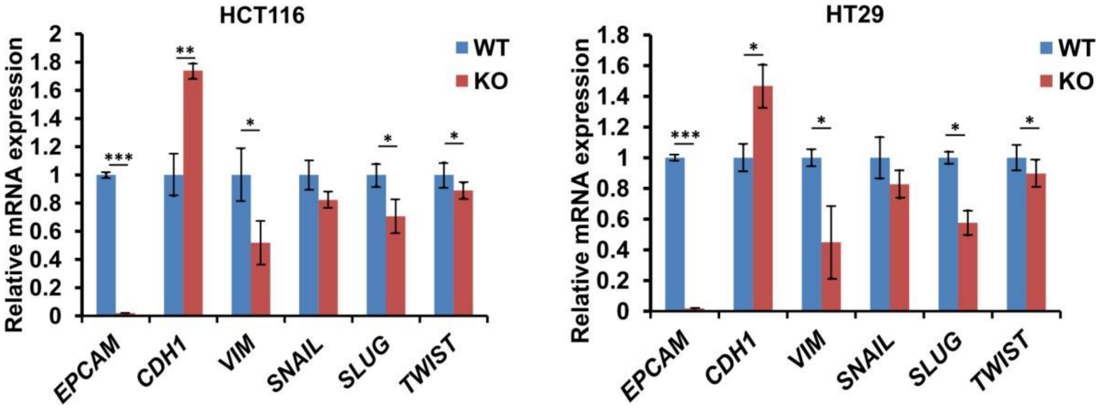

Fig. S5. EpCAM regulates EMT-related genes expression. The gene expression of EMT markers and regulators was detected by qRT-PCR in Wild-type (WT) or EpCAM knockout (KO) of HCT116 and HT29 cells. Statistical differences were determined by two-tailed Student *t* test. $N = 3$ independent experiments. All data are presented as mean ± SEM. *, $p < 0.05$; **, $p < 0.01$; ***, $p < 0.001$.

Fig. S7. Lee et al.

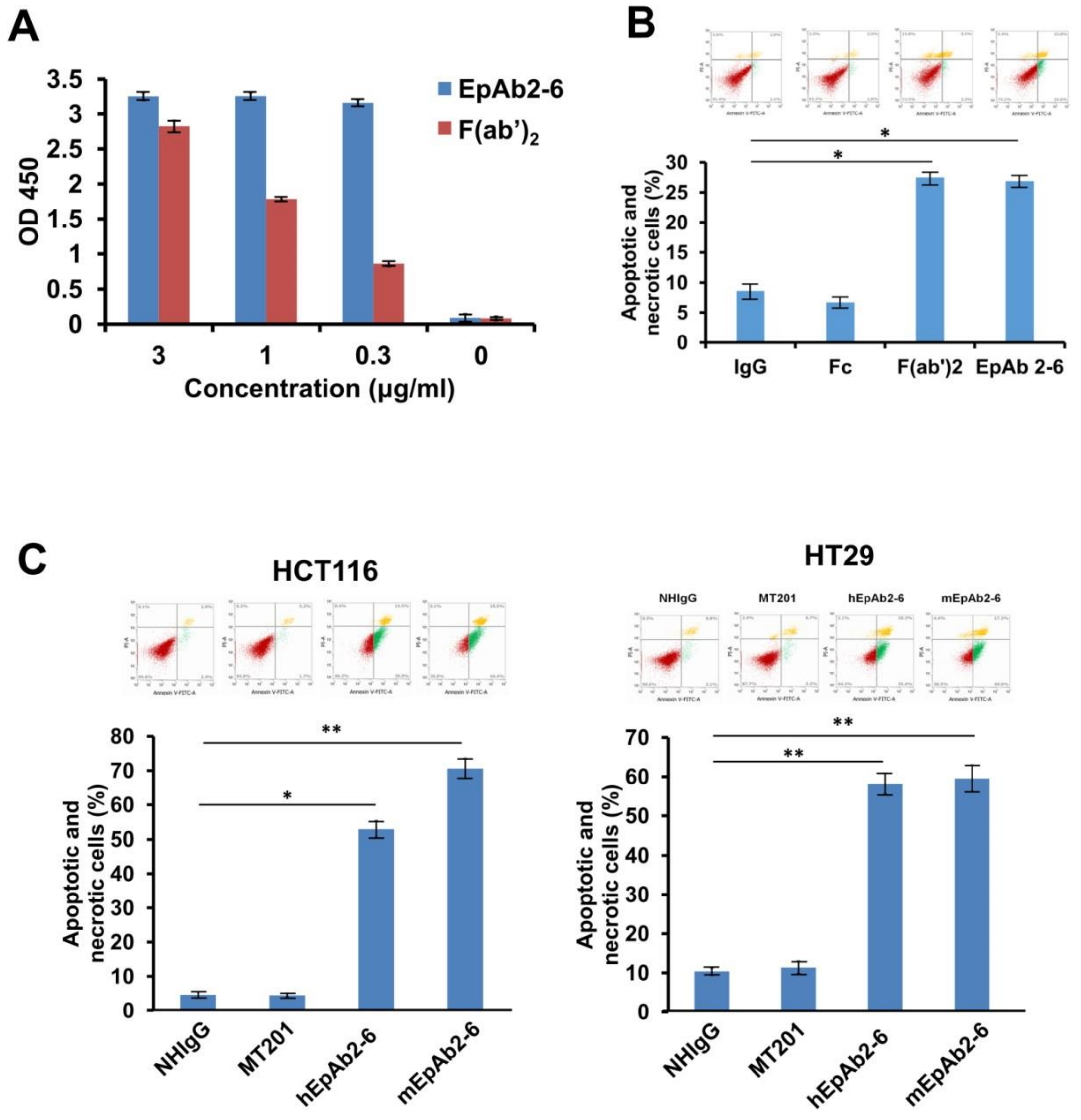

Fig. S6. EpAb2-6 binds to EpEX and induces apoptosis via F(ab')2.

(A) Binding affinity of IgG EpAb2-6 (mouse) and F(ab')$_2$ to EpEX-His (1µg/ml) coated overnight was checked using ELISA (OD450). (B) HCT116 cells were treated with 100 µg/ml control IgG, Fc, or F(ab')$_2$ of EpAb2-6 for 24h. The apoptotic and necrotic cells were quantified by fluorescein annexin V-FITC/PI double labeling. (C) HCT116 and HT29 cells were treated with 10 µg/ml control IgG, MT201, humanized EpAb2-6 (hEpAb2-6) or mouse hybridoma EpAb2-6 (mEpAb2-6) for 24h. The apoptotic and necrotic cells were quantified by fluorescein annexin V-FITC/PI double

labeling. Statistical differences were determined by two-tailed Student *t* test. $N = 3$ independent experiments. All data are presented as mean ± SEM. *, $p < 0.05$; **, $p < 0.01$.

Fig. S9. Lee et al.

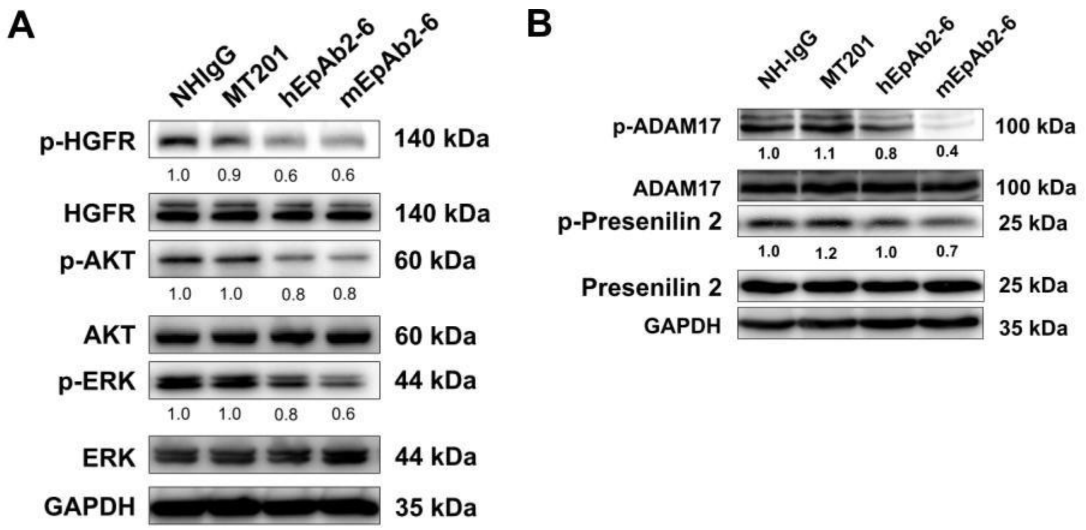

Fig. S7. EpAb2-6 inhibits regulated intramembrane proteolysis (RIP) activation and HGFR signaling. (A) HCT116 cells were treated with 10 μg/ml control IgG, MT201, hEpAb2-6 or mEpAb2-6 for 16h, followed by treatment with EpEX-His (50 nM) for 15 min. Levels of phosphorylated HGFR, AKT, and ERK, and (B) RIP proteins ADAM17 and presenilin 2 were examined by Western blotting. Anti-EpCAM antibody and crizotinib coordinately induces apoptosis in colon cancer cells.

Fig. S10. Lee et

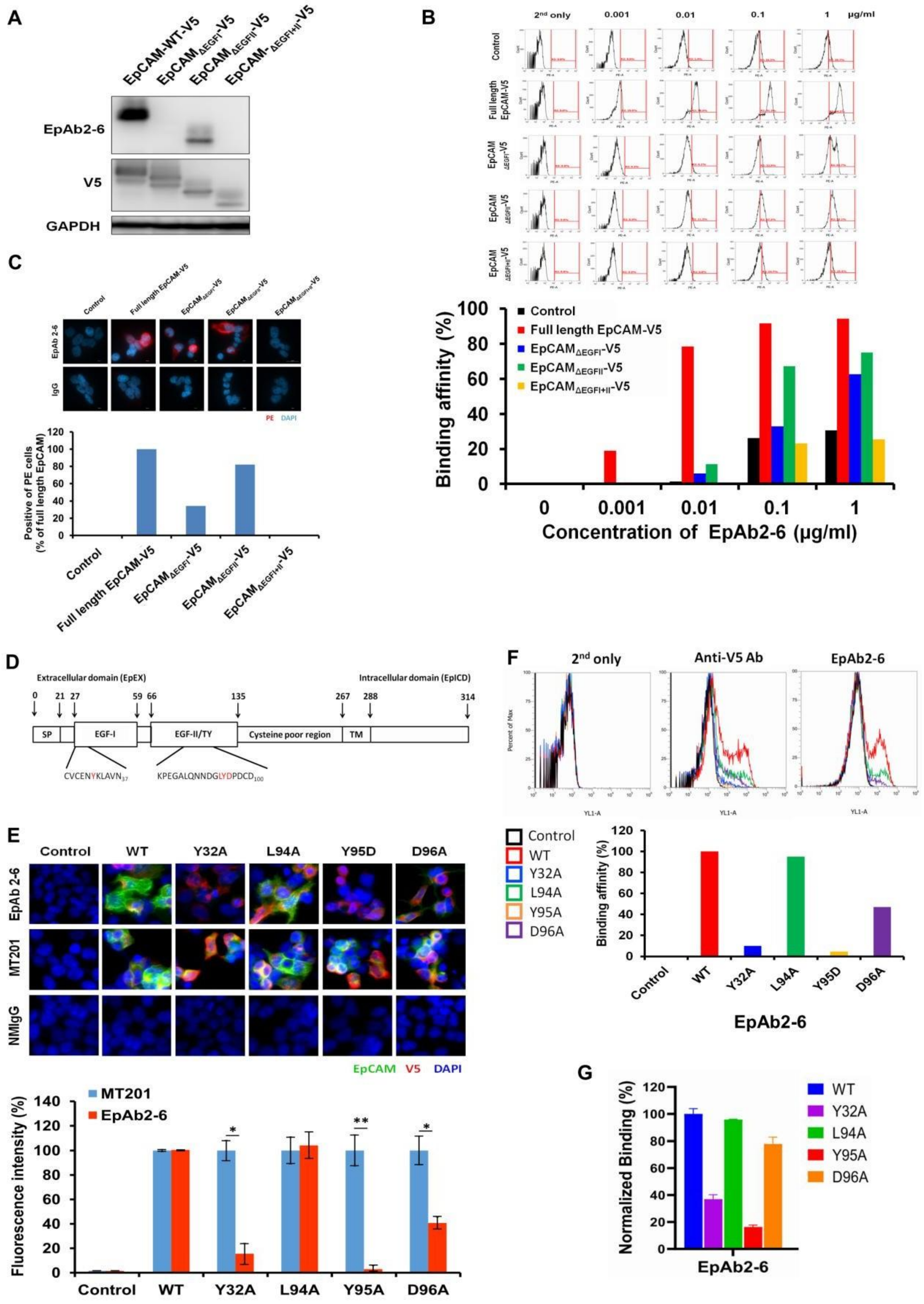

Fig. S 8. EpAb2-6 binds to both EGF-like domain I and II ofEpCAM. HEK293T cells were transfected with full length or EGF like-domain deletion mutant EpCAM-V5. Antibody binding was assessed by (A) Western blotting,

(B) flow cytometry, and (C) immunofluorescence. (D) EpCAM mutants were constructed with amino acid substitutions in the EGF-I (Y32A) and EGF-II (L94A, Y95A, or D96A) domains. EpCAM wild-type and mutant proteins were expressed in HEK293T cells. Binding of MT201 and EpAb2-6 to EpCAM wild-type and mutants were evaluated by (E) immunofluorescence, (F) flow cytometry, and (G) cellular ELISA. Statistical differences were determined by two-tailed Student t test. $N = 3$ independent experiments. All data are presented as mean ± SEM. *, $p < 0.05$; **, $p < 0.01$.

Fig. S9. Lee et al.

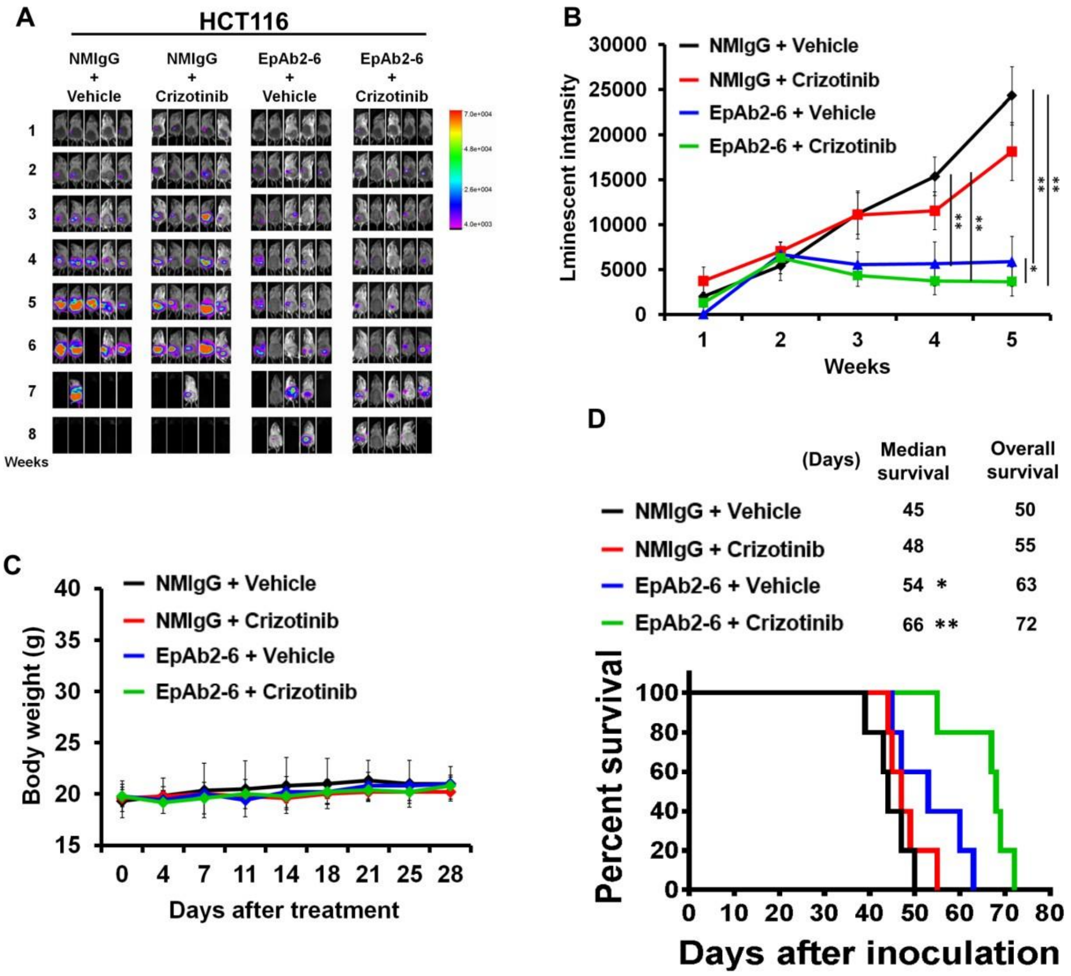

Fig. S9. EpAb2-6 and crizotinib coordinately inhibit tumor progression in the HCT116 orthotopic colon cancer animal model. (A) NOD/SCID mice received orthotopic implantation of HCT116-Luc cells and then were treated with control IgG (normal mouse IgG, NMIgG), crizotinib, EpAb2-6, or crizotinib combined with EpAb2-6 starting at 3 days after tumor inoculation. Tumor growth was monitored by examining bioluminescence with the IVIS 200 Imaging System. (B) HCT116-Luc tumor cells monitored by bioluminescence quantification.

(C) Body-weights of each treatment group in HCT116 orthotopic animal models after indicated treatments. (D) Survival curves and median survival days of each treatment group in HCT116 orthotopic animal models. Statistical differences were determined

by two-tailed Student t test. $N = 5$ independent experiments. All data are presented as mean ± SEM. *, $p < 0.05$; **, $p < 0.01$.

Table S1: List of oligonucleotides for real-time PCR assay

Gene	Sequence
EPCAM	Forward: 5'- CTCCACGTGCTGGTGTGT R -3'<br>Reverse: 5'- TGTTTTAGTTCAATGATGATCCAGTA -3'
CDH1	Forward: 5'- GGAACTATGAAAAGTGGGCTTG-3'<br>Reverse: 5'- AAATTGCCAGGCTCAATGAC-3'
VIM	Forward: 5'-GTTTCCCCTAAACCGCTAGG-3'<br>Reverse: 5'-AGCGAGAGTGGCAGAGGA-3'
SNAIL	Forward: 5'-CTTCGGCTCCAGGAGAGTC-3'<br>Reverse: 5'-TTCCCACTGTCCTCATCTGAC-3'
SLUG	Forward: 5'-ACACACACACACCCACAGAG-3'<br>Reverse: 5'-AAATGATTTGGCAGCAATGT-3'
TWIST	Forward: 5'-GGAGTCCGCAGTCTTACGAG-3'<br>Reverse: 5'-CCAGCTTGAGGGTCTGAATC-3'
GAPDH	Forward: 5'-CTTCACCACCATGGAGGAGGC-3'<br>Reverse: 5'-GGCATGGACTGTGGTCATGAG-3'
